# Performance evaluation of machine learning algorithms for predicting liquefaction-induced lateral displacement

**DOI:** 10.1038/s41598-026-50670-4

**Published:** 2026-04-30

**Authors:** Mahmood Ahmad, Mohammad Al Zubi, Shaikat Biswas, Abdur Rahman Rakib, Abdullah Alzlfawi, Sabahat Hussan, Shay Haq, Rohayu Che Omar, Zia Ullah, Zsolt Tóth

**Affiliations:** 1 Institute of Energy Infrastructure, Universiti Tenaga Nasional, Kajang, 43000 Malaysia; 2 Department of Civil Engineering, University of Engineering and Technology Peshawar (Bannu Campus), Bannu, 28100 Pakistan; 3 Department of Mechanical Engineering, Hijjawi Faculty for Engineering Technology, Yarmouk University, Irbid, Jordan; 4 Deparment of Computer Science, Troy University, 600 University Ave, Troy, AL 36082 USA; 5 Department of Civil and Environmental Engineering, College of Engineering, Majmaah University, Al Majmaah, 11952 Saudi Arabia; 6 Department of Civil Engineering, HITEC University, Taxila, 47040 Pakistan; 7 Department of Civil Engineering, National University of Sciences and Technology, Balochistan Campus, Quetta, 87300 Pakistan; 8 Broadridge Financial Solutions Inc, 51 Mercedes Way, Brentwood, NY 11717 USA; 9 Faculty of Wood Engineering and Creative Industries, University of Sopron, Sopron, Hungary

**Keywords:** Lateral displacement, Soil liquefaction, Machine learning, Performance measure, Engineering, Mathematics and computing, Natural hazards, Solid Earth sciences

## Abstract

Accurate prediction of liquefaction-induced lateral displacement is essential for seismic risk assessment, resilient infrastructure design, and cost-effective mitigation. Such predictions are complex and cannot be reliably addressed using conventional analytical approaches. This research utilizes XGBoost, CatBoost and AdaBoost algorithms with 247 post-liquefaction in-situ free-face ground condition case studies to model and investigate liquefaction induced lateral displacements. The models are assessed according to the coefficient of determination (R^2^), coefficient of correlation (*r*), mean absolute error (MAE), mean squared error (MSE), root means square error (RMSE), and Root Mean Squared Error-Observations standard deviation Ratio (RSR) and Nash Sutcliffe Efficiency (NSE) coefficient. The develop models are compared with each other, and also to the Gaussian process regression, artificial neural network, Evolutionary polynomial regression and Multiple linear regression models described in the literature. The XGBoost model had the best prediction performance, with R^2^ = 0.9905, *r* = 0.9952, MAE = 0.1491, MSE = 0.0485, RMSE = 0.2203, RSR = 0.0981, and NSE = 0.9904 for training and R^2^ = 0.9251, *r* = 0.9618, MAE = 0.3642, MSE = 0.3723, RMSE = 0.6101, RSR = 0.278, and NSE = 0.9227 for testing results respectively. The rank score analysis confirmed XGBoost to be superior, as it reached the highest total score of 40. Sensitivity analysis further revealed that T_15_ was the most sensitive parameter to output variable.

**Supplementary Information:**

The online version contains supplementary material available at 10.1038/s41598-026-50670-4.

## Introduction

Lateral displacement has been recognized as a particularly destructive type of ground failure in previous large-magnitude earthquake disasters^1^. Case studies show that liquefaction-induced lateral displacement is one of the principal causes of damage to pile foundations subjected to seismic stresses^2^. Liquefaction and its induced hazards such as lateral displacement can cause significant deformations and damage to existing structures, including ports, bridges, and pipelines^3–5^. Lateral displacement refers to the horizontal movement that occurs in gently inclined ground surfaces (typically with slopes less than 5%) when saturated soils experience excess pore water pressure or undergo liquefaction^1^. Lateral displacement can result in shear failure in pile foundations, causing surface structures to crack, strain, or even collapse^6^. Lateral displacement can cause numerous types of ground deformation, ranging in size from a few centimeters to several meters^7^. Lateral ground movement caused by liquefaction is a complicated geotechnical phenomenon that is governed by seismic loads, soil characteristics, and site conditions. As a result, conventional approaches for predicting such displacements frequently have intrinsic limits and cannot adequately reflect the complexity of the problem^8^. Several variables influence lateral displacement, including groundwater depth, the thickness and particle size of liquefiable soils, and the magnitude of the earthquake^7^. Several analytical, empirical, and computational models have been developed to anticipate how liquefiable soils will behave and how much lateral displacement may be triggered during seismic activity^3^. It is still difficult to adequately predict a liquefiable soil, despite intensive laboratory experimentation. These attempts are complicated by the challenge of getting “undisturbed” testing samples from the in situ deposit^7^. Predicting lateral displacements is a significant difficulty in geotechnical engineering because of the highly nonlinear interactions involved, which make it impossible to accurately handle using traditional methods. Therefore, using cutting-edge and creative assessment methodologies becomes crucial to designing practical and affordable mitigation strategies against liquefaction-induced lateral displacement^8^.

Liquefaction-induced lateral displacement has been examined through deformation monitoring and excavation-induced ground response analyses, providing insight into subsurface movement mechanisms relevant to seismic spreading^9^. Data-driven prediction of underground settlement, landslide formation mechanisms, hybrid displacement modeling, and nanoscale pore evolution collectively improve understanding of progressive ground deformation under instability^10–14^. Seismic detection techniques, discrete element simulations, tidal-stiffness profiling, seepage–erosion experiments, and intelligent risk models further enhance assessment of dynamic soil weakening and infrastructure vulnerability^15–19^. Advanced seismic interaction analyses, jacking-force prediction, unified critical state modeling, hydraulic control strategies, and seabed wave-response studies strengthen the theoretical and computational framework for evaluating liquefaction-driven lateral displacement in complex soil–structure systems^20–24^.

To assess the liquefaction induced lateral displacement, a number of analytical and empirical methods have been put forth. A useful and successful empirical tool among them is the Multiple Linear Regression (MLR) model, which was developed by regression analysis of recorded case histories^7^. Liquefaction-induced lateral displacement in earth dams, embankments, and slopes is made simple by an analytical method that combines Newmark’s sliding-block method with recorded acceleration time histories^25^. To assess the lateral displacement caused by liquefaction, a number of models have been developed using the finite element method. Gu and Robertson^26^ evaluated the Lower San Fernando Dam’s post-earthquake deformations using an incremental finite element method. Gu and Robertson^27^ applied a plane-strain model to analyze liquefaction-induced deformations, successfully capturing the displacement pattern at a wildlife site in California, though the displacements were overestimated by approximately 30%. In contrast, a stochastic and non-parametric method called Gaussian Process Regression (GPR) has become a viable machine learning (ML) strategy for dealing with challenging and nonlinear issues^28^.

In recent years, ML have received growing attention in civil engineering for their ability to address complex tasks, where conventional deterministic methods remain limited^29–38^. The application of AI methods has increased considerably, primarily due to their ability to model and predict highly complex non-linear interactions^29,30,39–41^. ML is an emerging set of diverse techniques that tend to reconcile the low precision and uncertainty as well as the lack of information using some statistical, probabilistic, and optimization tools in order to analyze sets of data, categorize the information, discover novel patterns and predict forthcoming trends with the briefest delay^42^. By gradually integrating several weak learners, the gradient boosting technique creates a powerful prediction model. In order to minimize total loss, these learners are connected in phases during training. The model iteratively modifies the weights at each stage to lower the error between the predicted and observed values^43^. In contrast to models like random forest or support vector machine, the algorithms Extreme Gradient Boosting (XGBoost), Adaptive Boosting (AdaBoost), and Categorical Boosting (CatBoost) were chosen for their superior accuracy and computational efficiency, as well as their capacity to handle high-dimensional data and capture non-linear relationships^44–46^. The frequent usage of these algorithms in research across fields demonstrates their versatility and adaptability to a wide range of domains; nevertheless, literature reviews indicate that their applications in seismic liquefaction-induced hazards are limited.

This study’s primary contributions are: (1) development of novel predictive models for liquefaction-induced lateral displacement with high precision and accuracy; (2) rigorous validation of the proposed models through comparative performance assessment against established models in the literature; and (3) execution of a sensitivity analysis to identify the most influential input parameters.

## Data catalog and analytics

This study’s dataset includes 247 free-face lateral displacement recordings obtained from Youd et al.^47^, Cetin et al.^48^, and Chu et al.^49^(see Supplementary information file, Table S1). Regarding data quality, the dataset was thoroughly screened prior to analysis, with no missing values identified. Additionally, the data distribution was carefully examined to confirm the absence of significant outliers, ensuring the integrity and robustness of the model training process. These factors support the reliability of the reported evaluation metrics and overall model performance. The dataset was adopted from published case histories in a consistent form; therefore, no additional pre-processing or normalization was applied. This study used seven major input parameters to evaluate lateral displacement (D_H_): earthquake magnitude (M), attenuation-based peak ground acceleration (*a*_*max*_) estimates derived from the ground-motion relationships of Sadigh et al.^50^, horizontal distance from the seismic energy source (*R*), mean particle size within T_15_ (D50_15_), cumulative thickness of saturated layers with corrected SPT values (N_1_)_60_ < 15 (T_15_), and average fines content (< 0.075 mm) in T_15_ (F_15_). These input parameters were selected based on key variables consistently reported in previous numerous studies that are widely accepted for predicting lateral displacement (e.g., Ahmad et al.^28^, Javadi et al.^51^, and Jafarian and Nasri^52^. This study incorporates peak ground acceleration (PGA, *a*_*max*_) as an additional intensity measure, estimated using fault-mechanism-based attenuation model by Sadigh et al.^50^ to improve prediction accuracy.

The dataset of 247 case histories was partitioned into training and testing subsets. The training set, comprising 198 samples (80% of the data), was used to develop the model, while the testing set, consisting of 49 samples (20% of the data), was employed to assess its performance. The statistical distributions of both input and output parameters for the two subsets are presented in Table 1.

**Table 1:** Statistical Parameters for free face condition.

Dataset	Parameter								Output
	Statistical	Seismic			Geotechnical			Topographic	
		M	R (km)	*a*_*max*_ (g)	F_15_ (%)	D50_15_ (mm)	T_15_ (m)	W (%)	D_H_ (m)
Training	Minimum	6.4	0.5	0.15	1	0.04	0.2	1.64	0
	Average	7.26	15.34	0.4	17.25	0.39	8.22	11.54	2.49
	Maximum	9.2	100	0.68	70	7.7	16.7	57.7	10.16
	Standard Deviation	0.48	11.03	0.14	13.06	0.67	5.1	10.34	2.25
Testing	Minimum	6.4	0.5	0.15	3	0.07	0.5	2.11	0
	Average	7.27	15.12	0.42	20.33	0.27	6.27	10.63	2.03
	Maximum	9.2	60	0.68	66	1.47	15.6	48.98	9.29
	Standard Deviation	0.62	12.93	0.16	15.18	0.29	5.13	8.08	2.22

To assess the significance of associations between variables, correlation coefficients (ρ) were calculated. The parameters showed moderate to weak correlations, and the largest absolute correlation coefficient (|ρ|) was < 0.8 (see Table 2). It was also tested whether multicollinearity could have played a role, since highly correlated predictor variables can inflate rather than decrease variance, and it may be difficult to detect true effects of individual predictors. The outcomes showed that some parameters were moderately associated (e.g., M–R, R–*a*_*max*_) but all the results did not surpass the generally accepted benchmark of multicollinearity (|ρ| > 0.8)^53^. Therefore, multicollinearity was unlikely and did not negatively affect the performance of the model.

**Table 2:** Input and output parameters correlation.

Parameters	M	*R*	a_max_	F_15_	D50_15_	T_15_	W	D_H_
		(km)	(g)	(%)	(mm)	(m)	(%)	(m)
M	1							
R (km)	0.76	1						
*a*_max_ (g)	−0.34	−0.72	1					
F_15_ (%)	−0.37	−0.37	0.56	1				
D50_15_ (mm)	0.03	0.01	−0.11	−0.23	1			
T_15_ (m)	0.21	0.36	−0.57	−0.59	0.24	1		
W (%)	0.00	−0.05	0.18	0.25	0.03	−0.15	1	
D_H_ (m)	0.18	0.23	−0.25	−0.35	−0.08	0.52	0.15	1

**Fig. 1:**
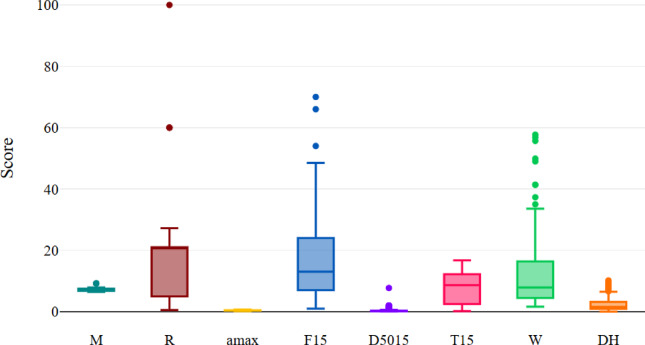
Box plot distribution of liquefaction-induced lateral displacement parameters.

The box plot distribution of liquefaction-induced parameters was plotted as presented in Fig. 1. The findings reveal that the magnitude of earthquake (M) and peak ground acceleration (*a*_*max*_) has low dispersion, which helps in expressing the similarities in case histories of seismic input conditions. In comparison, horizontal distance to seismic energy source (R) records highest variation and positive skew, implying that there is a wide variation of values in the dataset in connection to horizontal distance to seismic energy source. Average fines (particles < 0.075 mm) in T_15_ (F_15_) and free-face ratio (W) also have significant variability and high value outliers and hence indicate that there is strong variability in the soil composition and free-face conditions. Parameters, like average particle size in T_15_ (D50_15_), and accumulative thickness of saturated layers with adjusted SPT number (N_1_)_60_ < 15 (T_15_), are considered to have relatively compact distributions, which suggest some more even site-specific geotechnical properties. Parameters i.e., R, F_15_ and W are highly variable, which shows their significant impact on spatial variability of liquefaction manifestation.

## Methodology

### Adaptive boosting (AdaBoost)

With a strong theoretical foundation and a proven track record of effectiveness in real-world applications, AdaBoost is among the excellent boosting algorithms as it introduces an innovative and new concept to learning algorithm creation by using a weak learning algorithm with accuracy only marginally better than random guessing to create an arbitrarily accurate strong learning algorithm^45^. The goal of the machine learning technique known as “boosting” is to combine several, incorrect, and weak rules to produce a single, highly accurate prediction rule^54^.

1 $${\mathrm{Final}}\;{\mathrm{Hypothesis}},F\left( x \right) =\sum {a_t}{h_t}\left( x \right)$$

where

weak hypothesis = *h*(*x*).

2 $$\alpha\:t\hspace{0.17em}=\hspace{0.17em}\frac{1}{2}\mathrm{l}\mathrm{n}(\frac{1-{\boldsymbol{\upepsilon\:}}_{\mathbf{t}}\:}{{\boldsymbol{\upepsilon\:}}_{\mathbf{t}}\:}$$

The weak learner aims to identify a weak hypothesis that has a low weighted error, *ε*_*t*_, in relation to weak learner distribution. The combined or final hypothesis determines the value of a weighted set of weak hypotheses^54^.

### Extreme gradient boosting (XGBoost)

Sequential decision trees are produced by the enhanced gradient tree boosting technique known as XGBoost. In all computing environments, it can swiftly do correlated calculations^55^. XGBoost employs a gradient boosting framework, sequentially constructing trees based on the residuals of preceding iterations, while incorporating regularization terms into its objective function to mitigate overfitting and enhance both generalization and predictive performance^56^. Through hardware optimization, tree pruning, and parallelization, the XGBoost method enhances the system’s performance^55^.

### Categorical boosting (CatBoost)

CatBoost is a ML ensemble technique that belongs to the GBDT family^58^. CatBoost is a gradient boosting method that makes use of binary decision trees as its key predictor^59^. Ordered target encoding is the basic concept of CB’s preprocessing of categorical features. It works by randomly sorting the dataset and then only using the objects that came before the current object to compute the categorical feature’s numerical conversion^60^. In the event where the *k*^th^ sample’s *i*^th^ feature is a categorical feature, the conversion formula can be written as

3 $$\:{\boldsymbol{x}\:}_{\boldsymbol{k}}^{\boldsymbol{i}}=\frac{\sum\:{\boldsymbol{x}}_{\boldsymbol{j}}\in\:{\boldsymbol{D}}_{\boldsymbol{K}}\:\{{\boldsymbol{x}\:}_{\boldsymbol{k}}^{\boldsymbol{i}}\:=\:{\boldsymbol{x}\:}_{\boldsymbol{j}}^{\boldsymbol{i}}\}\:\times\:\:{\boldsymbol{y}}_{\boldsymbol{j}}\:+\:\boldsymbol{a}\:\times\:\:\mathbf{p}}{\sum\:{\boldsymbol{x}}_{\boldsymbol{j}}\in\:{\boldsymbol{D}}_{\boldsymbol{K}}\:\{{\boldsymbol{x}\:}_{\boldsymbol{k}}^{\boldsymbol{i}}\:=\:{\boldsymbol{x}\:}_{\boldsymbol{j}}^{\boldsymbol{i}}\}\:+\:\boldsymbol{a}\:}$$

where *p* is the added prior item, ‘*a*’ is usually a weighting coefficient greater than 0. *D*_*k*_ is the dataset before the *k*^th^ sample in the random ordering, and $\:\{{x\:}_{k}^{i}\:=\:{x\:}_{j}^{i}\}\:$=1 when $\:{x\:}_{k}^{i}$ and $\:{x\:}_{j}^{i}$ belong to the same category and $\:\{{x\:}_{k}^{i}\:=\:{x\:}_{j}^{i}\}\:$=0 when they belong to different categories^60^.

### Performance evaluation

The evaluation stage involves the computation of diverse assessment metrics, encompassing coefficient of determination (R^2^), root mean squared error (RMSE), mean absolute error (MAE), root mean square standard deviation ratio (RSR), and Nash-sutcliffe efficiency (NSE). These metrics serve to gauge the efficacy of the model’s performance, shedding light on the extent to which the model’s predictions correlate with the actual target values. The formulations used to calculate these performance metrics are expressed in Eqs. (4)–(10)^61–67^.

4 $$\:{\mathrm{R}}^{2}=1-\frac{{\sum\:}_{\mathrm{i}=1}^{n}{\left({d}_{i}-\left.{y}_{i}\right)\right.}^{2}}{{\sum\:}_{i=1}^{n}{\left({d}_{i}-\left.{d}_{mean}\right)\right.}^{2}}\:$$

5 $$r=\frac{{\sum\nolimits_{{i=1}}^{n} {\left( {{d_i} - {d_{mean}}} \right)\left( {{y_i} - {y_{mean}}} \right)} }}{{\sqrt {\sum\nolimits_{{i=1}}^{n} {{{\left( {{d_i} - {d_{mean}}} \right)}^2} \cdot \sum\nolimits_{{i=1}}^{n} {\left( {{y_i} - {y_{mean}}} \right)} } } }}$$

6 $$\:RMSE=\sqrt{\frac{1}{n}{\sum\:}_{i=1}^{n}{\left({d}_{i}-\left.{y}_{i}\right)\right.}^{2}}$$

7 $$MSE=\frac{1}{n}\sum\limits_{{i=1}}^{n} {{{\left( {{d_i} - {y_i}} \right)}^2}}$$

8 $$\:MAE=\frac{1}{n}{\sum\:}_{i=1}^{n}\left|\left({y}_{i}-\left.{d}_{i}\right)\right.\right|$$

9 $$RSR=\frac{{\sqrt {\sum\nolimits_{{i=1}}^{n} {{{\left( {{d_i} - {y_i}} \right)}^2}} } }}{{\sqrt {\sum\nolimits_{{i=1}}^{n} {{{\left( {{d_i} - {d_{mean}}} \right)}^2}} } }}$$

10 $$NSE=1 - \frac{{\sum\nolimits_{{i=1}}^{n} {{{\left( {{d_i} - {y_i}} \right)}^2}} }}{{\sum\nolimits_{{i=1}}^{n} {{{\left( {{d_i} - {d_{mean}}} \right)}^2}} }}$$

where *d*_*i*_ is the *i*th observed value, *y*_*i*_ is the *i*th predicted value, *d*_*mean*_ is the mean value of the observed values, *y*_*mean*_ is the mean value of the predicted values and *n* is the number of data points.

The optimal model exhibits R^2^, *r*, and NSE values close to 1, with minimal RMSE, MSE, MAE, and RSR approaching 0.

## Model development and evaluation

The data set of 247 post-liquefaction in-situ free-face ground condition case studies were divided into training (80%) and testing (20%) sets to maintain the statistical consistency of all the input variables. AdaBoost, XGBoost and CatBoost algorithms were used to create three models based on the training dataset and tested on the testing dataset. The parameter range was predefined and used a random search strategy to tune hyperparameters, which was used only on the training subset to achieve the best results as indicated in Table 3. The early stopping criteria is used to stop training when the model’s performance i.e., root mean squared error (RMSE) value on a testing set no longer improves or starts to degrade. It helps prevent overfitting by halting the training process before the model starts to memorize the noise in the data. The performance of the model was tested based on seven statistical parameters: R^2^; *r*; MAE; MSE; RMSE; RSR ratio and NSE coefficient.

**Table 3:** Optimal hyperparameters and the search range.

Algorithm	Hyperparameter	Value	Range
XGBoost	Number of trees	70	50–200
	Learning rate	0.162	0.01–0.3
	Lambda (L2 regularization)	1	0–5
	Maximum tree depth	7	3–10
	Subsample (fraction of training instances)	0.6	0.5–1.0
	Colsample_bytree (fraction of features per tree)	1	0.5–1.0
	Colsample_bylevel (fraction of features per level)	1	0.5–1.0
	Colsample_bynode (fraction of features per split)	0.4	0.3–1.0
CatBoost	Number of trees	69	50–200
	Learning rate	0.268	0.01–0.3
	Lambda (L2 regularization)	3	0–5
	Maximum tree depth	6	3–10
	Colsample_bytree (fraction of features per tree)	1	0.5–1.0
AdaBoost	Base estimator	Decision Tree	–
	Number of estimators	181	50–250
	Learning rate	1	0.01–2.0
	Classification algorithm	SAMME	–
	Regression loss function	Square	–

## Results and discussions

### Performance of AdaBoost model

**Fig. 2:**
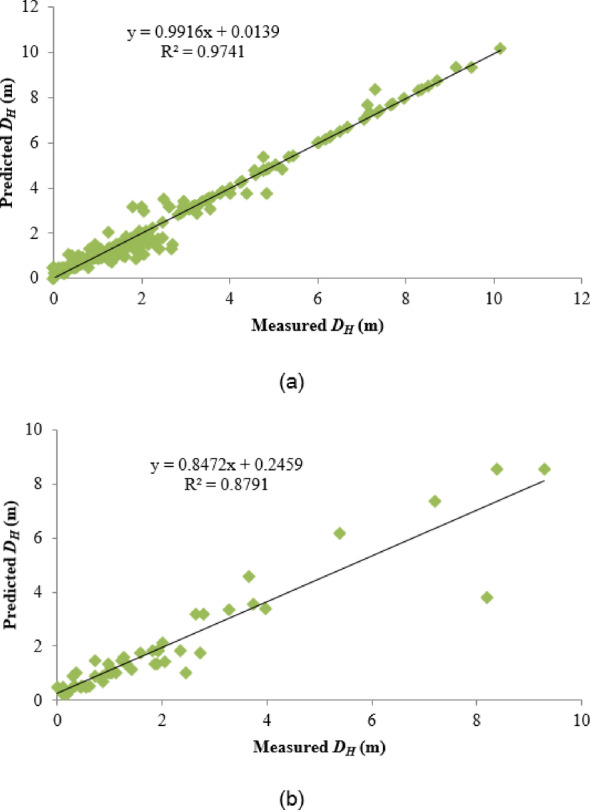
Scatter plot of AdaBoost model (**a**) training and (**b**) testing datasets.

**Fig. 3:**
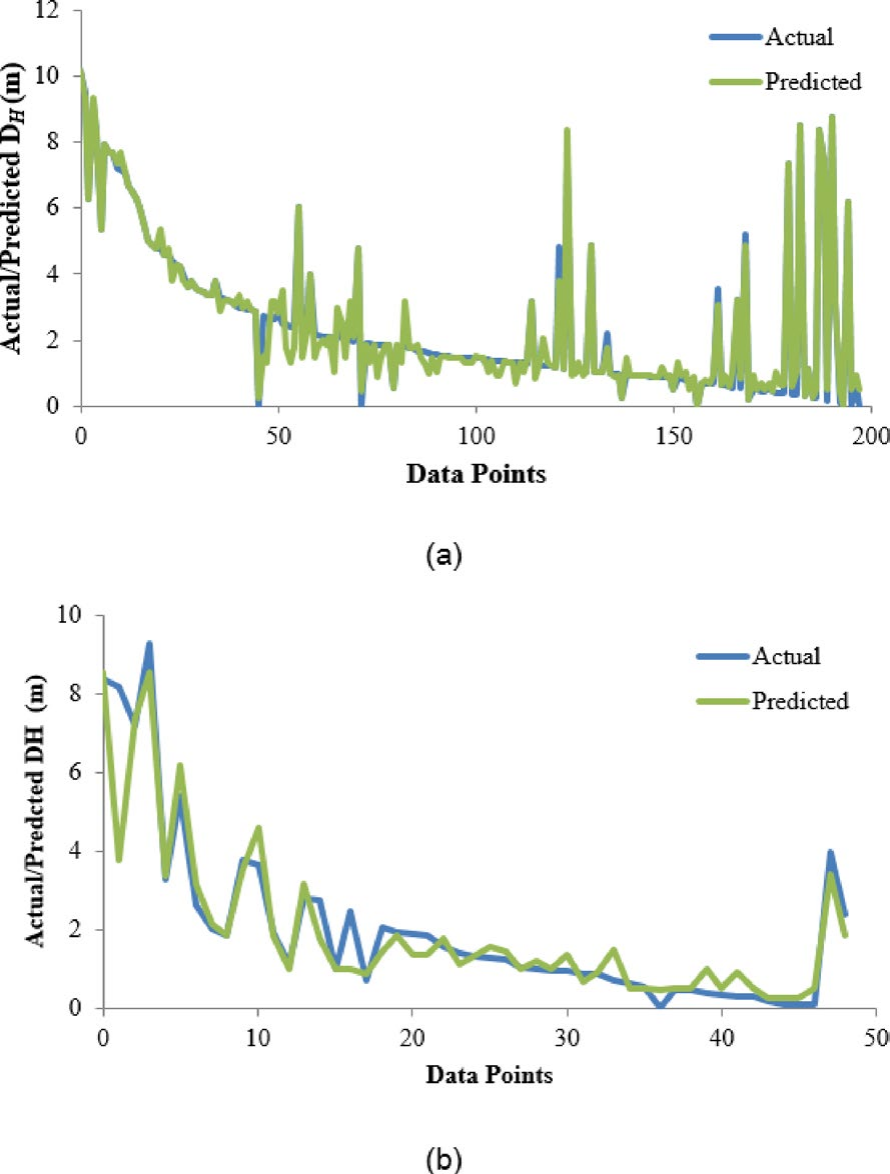
Line graph of AdaBoost model showing the actual D_H_ values against predicted D_H_ (**a**) training and (**b**) testing datasets.

**Fig. 4:**
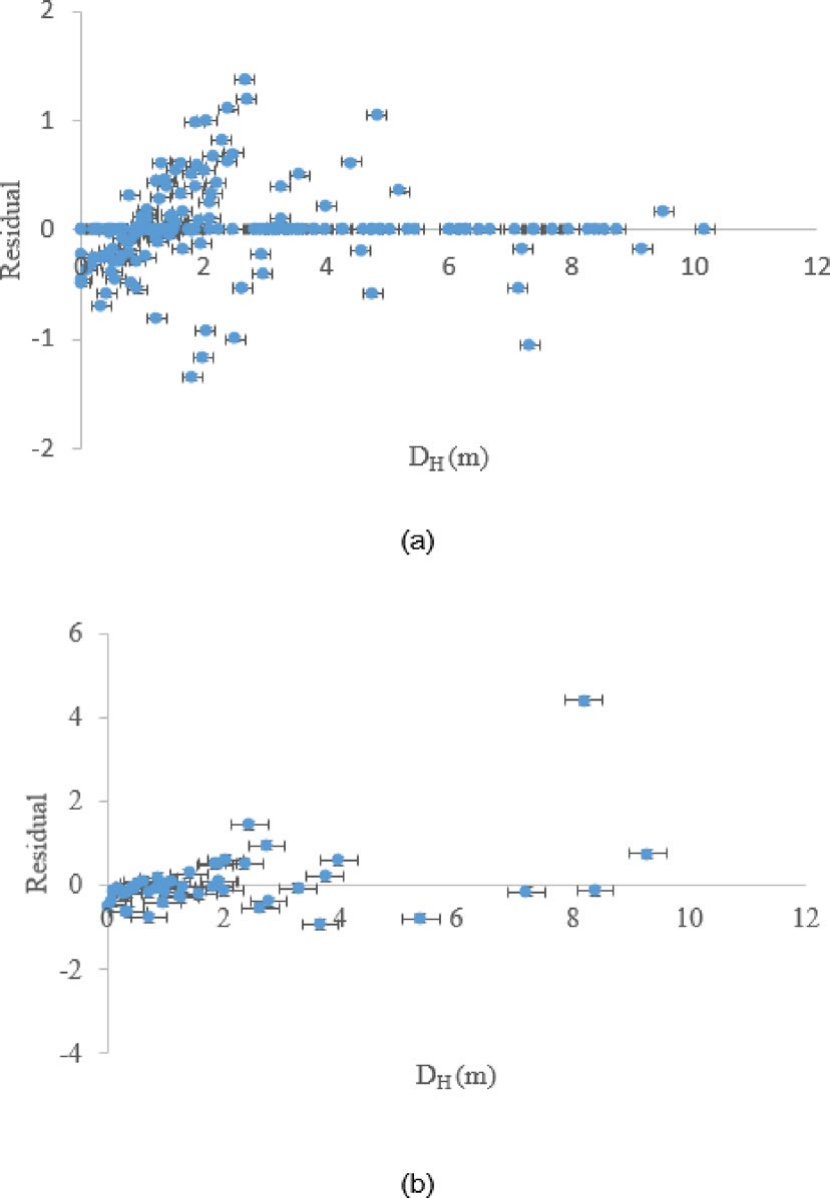
Residual error plot of the AdaBoost model for (**a**) training and (**b**) testing datasets.

Coefficient of determination (R^2^), correlation coefficient (*r*), mean absolute error (MAE), root mean square error (RMSE), ratio of RMSE to standard deviation of observed value (RSR) and Nash-Sutcliffe coefficient (NSE) were employed to assess the accuracy and precision of AdaBoost model. The trend line for AdaBoost model, for both testing and training phase respectively is plotted by comparing the actual regression, depicted in Figs. 2 and 3. The AdaBoost model shows a comparatively larger error dispersion in both training and testing datasets, with clear deviations from the zero-error line, according to the residual error plots in Fig. 4. This implies that while the model captures broad patterns, it is inconsistent and may exhibit moderate prediction bias, especially for data that has for unseen data.

Table 4 shows obviously that R^2^ =0.9741, *r* = 0.987, MAE = 0.2037, MSE = 0.1324, RMSE = 0.3638, RSR = 0.162 and NSE = 0.9737 for the training dataset whereas R^2^ =0.8791, *r* = 0.9376, MAE = 0.4501, MSE = 0.5918, RMSE = 0.7693, RSR = 0.3506 and NSE = 0.8771 for the testing dataset.

### Performance of CatBoost model

**Fig. 5:**
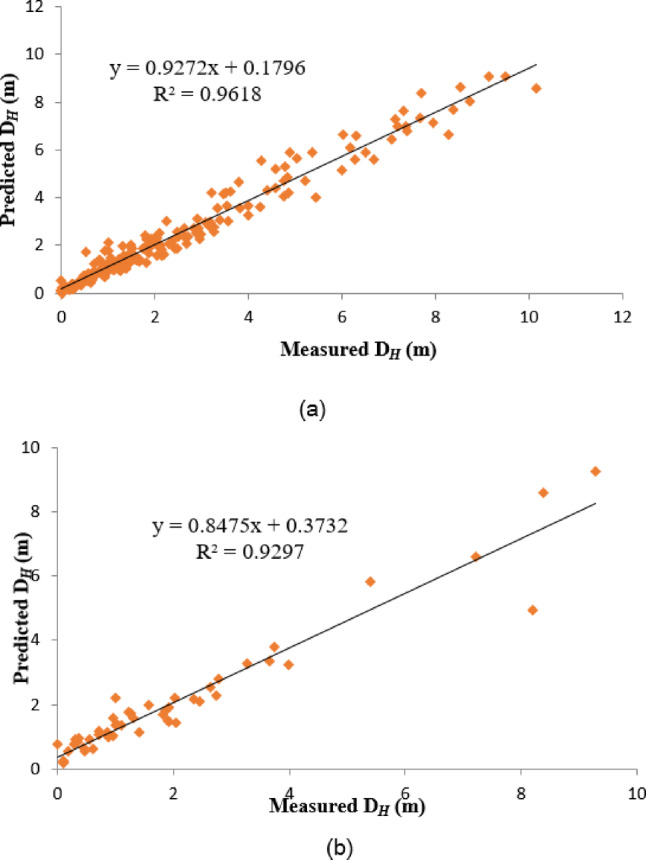
Scatter plot of CatBoost model (**a**) training and (**b**) testing datasets.

**Fig. 6:**
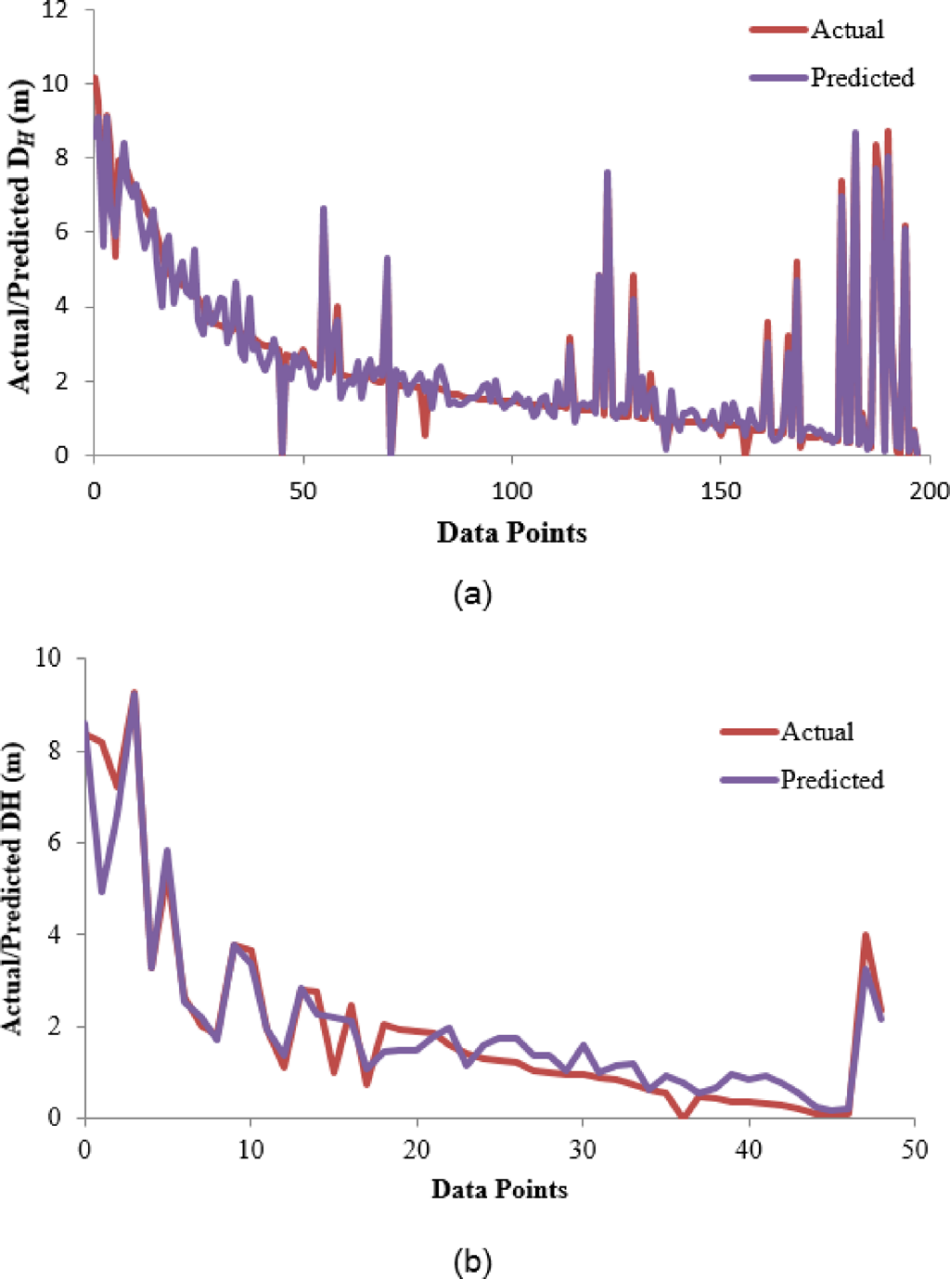
Line graph of CatBoost model presenting the actual *D*_*H*_ values versus the predicted D_H_ (**a**) training and (**b**) testing datasets.

**Fig. 7:**
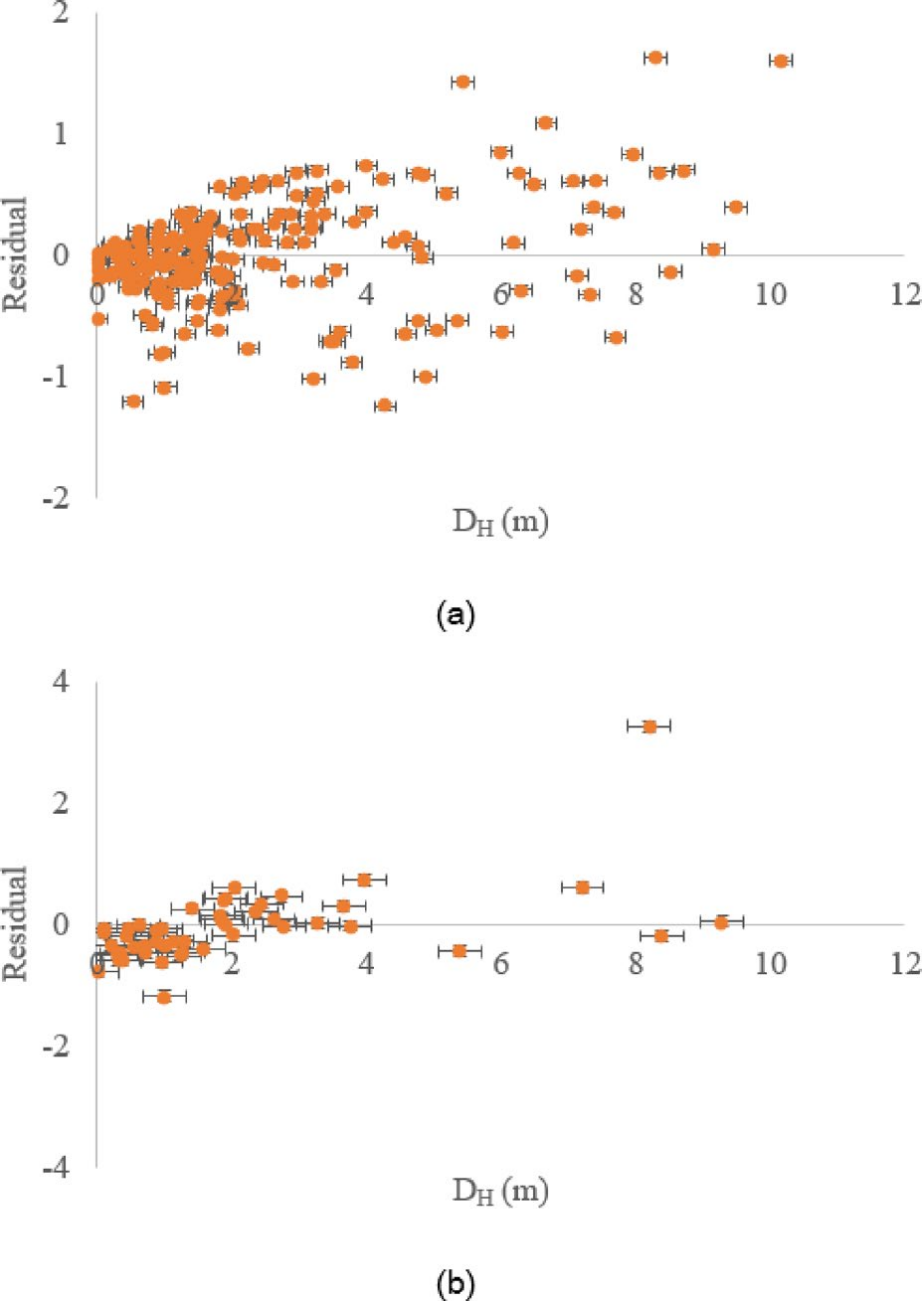
Residual error plot of the CatBoost model for (**a**) training and (**b**) testing datasets.

The graphs of actual and predicted data for testing and training datasets of CatBoost model have plotted in Figs. 5 and 6. For training model R^2^ = 0.9618, *r* = 0.9807, MAE = 0.3272, MSE = 0.1988, RMSE = 0.4461, RSR = 0.1987 and NSE = 0.9605 and for testing model R^2^ = 0.9297, *r* = 0.9642, MAE = 0.3845, MSE = 0.0485, RMSE = 0.6145, RSR = 0.28 and NSE = 0.9216. In comparison to AdaBoost, the CatBoost model exhibits better residual clustering around zero, as seen in Fig. 7; nonetheless, considerable spread and outliers remain, particularly during the testing stage. Although there is still some variation in its generalization performance, this indicates improved learning capacity and decreased bias.

### Performance of XGBoost model

**Fig. 8:**
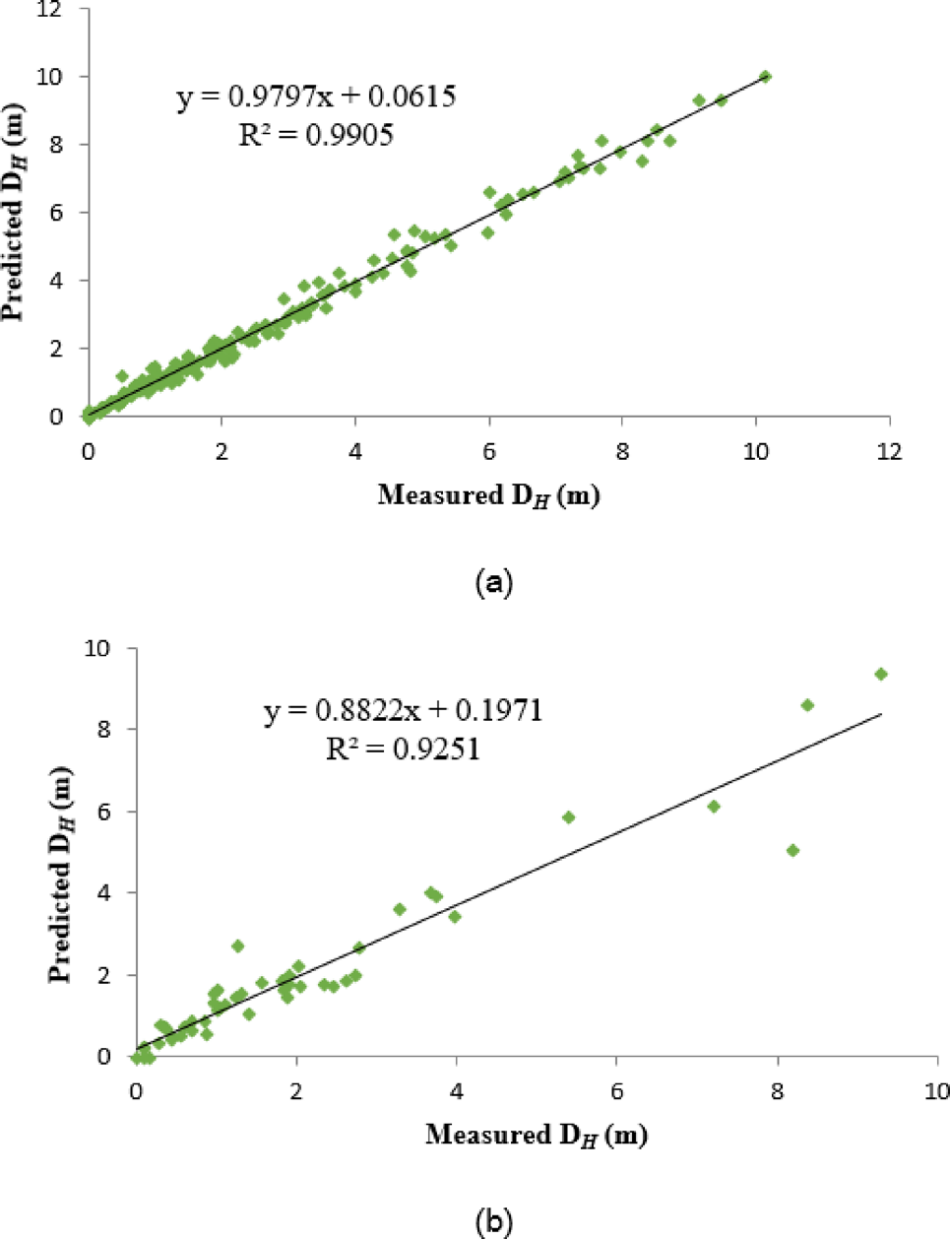
Scatter plot of XGBoost model (**a**) training and (**b**) testing datasets.

**Fig. 9:**
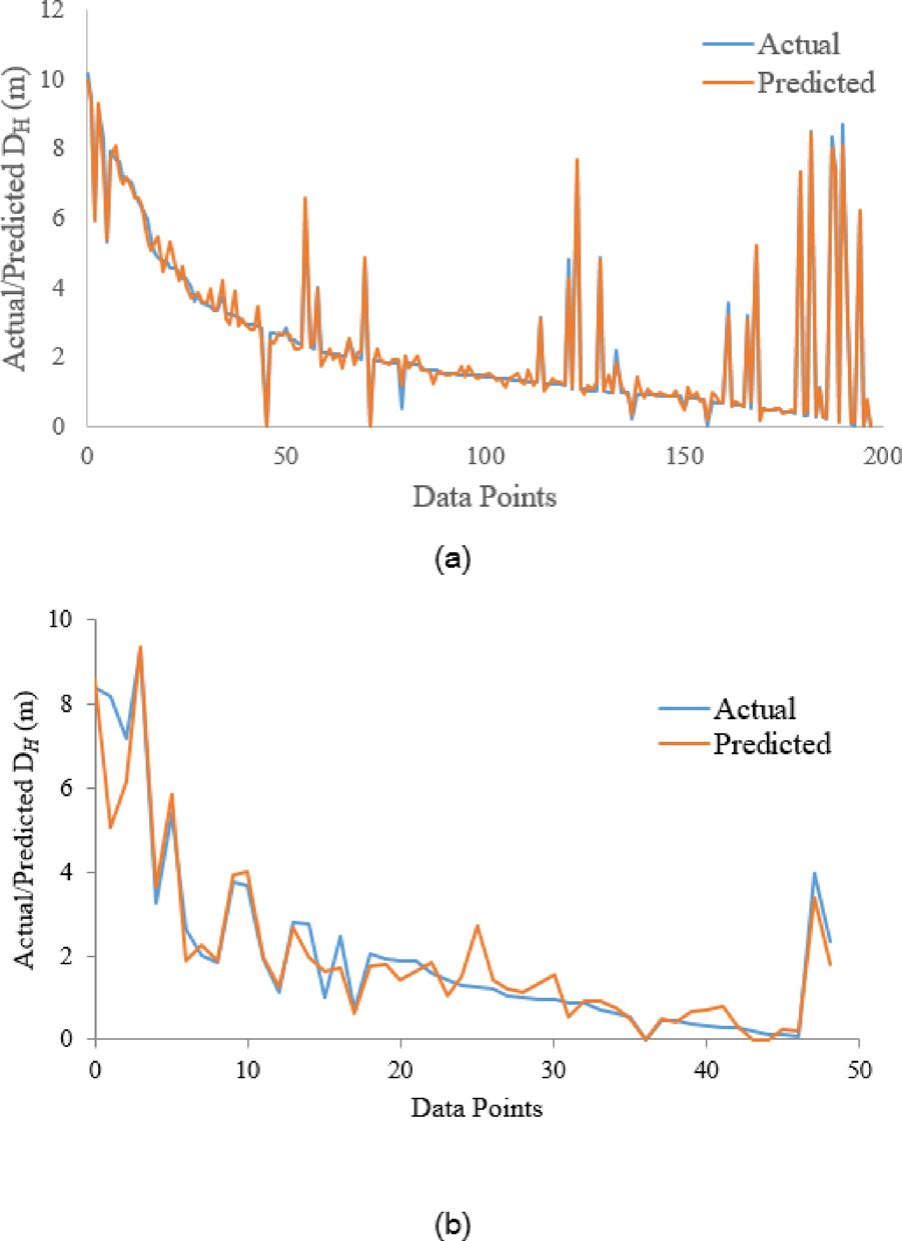
Line graph of the XGBoost model presenting the actual *D*_*H*_ values versus the predicted *D*_*H*_ (**a**) training and (**b**) testing datasets.

**Fig. 10:**
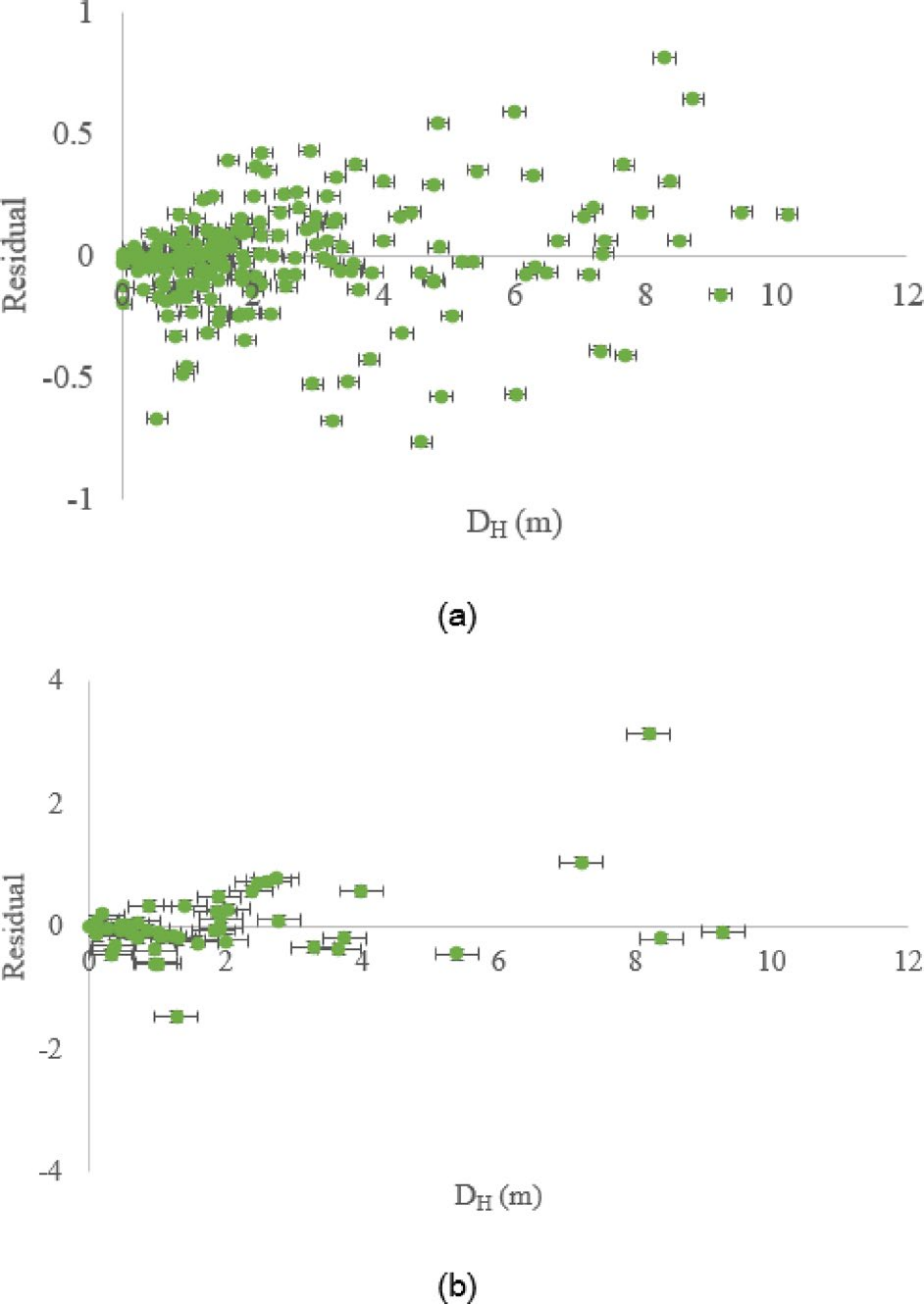
Residual error plot of the XGBoost model for (**a**) training and (**b**) testing datasets.

The trend line is provided for the observed regression, R^2^ = 0.9905 for training model and R^2^ = 0.9251 for testing XGBoost model (see Figs. 8 and 9). It could be noted from Table 4, for the training XGBoost model with *r* = 0.9952, MAE = 0.1491, MSE = 0.0485, RMSE = 0.2203, RSR = 0.0981 and NSE = 0.9904 and for the XGBoost model in testing dataset with *r =* 0.9618, MAE = 0.3642, MSE = 0.3723, RMSE = 0.6101, RSR = 0.278 and NSE = 0.9227. Figure 10 shows that the XGBoost model’s residuals around the zero line are more compact and uniformly distributed on both the training and testing datasets. The lower dispersion and fewer outliers suggest improved prediction accuracy, resilience, and excellent generalizability. According to the analysis of Figs. 4 and 7, and 10, the XGBoost model outperforms AdaBoost and CatBoost by having the most consistent and least dispersed residuals, indicating it as the most dependable and accurate model for predicting D_H_ in the study.

### Performance evaluation with literature models

In the testing phase, the performance of the developed ensemble learning models—AdaBoost, CatBoost, and XGBoost—was evaluated and compared with existing literature models using key statistical indicators, including R^2^, *r*, MAE, MSE, RMSE, RSR, and NSE. As in Table 4, the performance of XGBoost was most favorable as it scored an R^2^ of 0.9251, *r* of 0.9618, MAE of 0.3642, MSE of 0.3723, RMSE of 0.6101, RSR of 0.278, and NSE of 0.9227. These findings show great predictive reliability and show great concordance with observed values. CatBoost closely came next with highest R^2^ (0.9297) and *r* (0.9642), indicating slightly better fit and correlation with observed data, yet has slightly high prediction errors (MAE = 0.3845, MSE = 0.3772, RMSE = 0.6145, RSR = 0.280, NSE = 0.9216) than the XGBoost. Conversely, AdaBoost produced the worst result on all the measures with R^2^ = 0.8791, *r* = 0.9376, MAE = 0.4051, MSE = 0.5918, RMSE = 0.7693, RSR = 0.3506 and NSE = 0.8771, reflecting lower predictive capability. The results of testing dataset (see Table 4) demonstrated that XGBoost produced the greatest predictive accuracy with R^2^ = 0.9905 (training) and 0.9251 (testing), correlation coefficients (*r*) = 0.9952 and 0.9618, and the error metrics (MAE = 0.1491, MSE = 0.0485, RMSE = 0.2203 in training; MAE = 0.3642, MSE = 0.3723, RMSE = 0.6101 in testing) as compared to the Gaussian Process Regression (GPR) with (R^2^ = 0.9402/0.894, RMSE = 0.5597/0.8438 in training/test) against EPR (R^2^ = 0.913/0.883, RMSE = 1.003/1.157), ANN (R^2^ = 0.875/0.872, RMSE = 1.074/1.21 and MLR (R^2^ =0.868/0.875, RMSE = 1.24/1.196) in literature. For XGBoost, it was observed a slight gap between training and testing performance, indicating slight overfitting; this is likely due to its high model complexity and sensitivity to hyperparameter settings. Regarding AdaBoost, its comparatively weaker testing performance can be attributed to its sequential weighting mechanism, which makes it more sensitive to noisy data and outliers, reducing generalization compared to XGBoost and CatBoost. Overall, the ensemble models, particularly the XGBoost, showed superior predictive ability and generalization during the testing, compared with all the baseline models across all the evaluation metrics including, R^2^, *r*, MAE, MSE, RMSE, RSR, and NSE.

**Table 4:** Performance statistics of models in comparison with extra available models in literature.

Model	Dataset	*R* ^2^	*r*	MAE	MSE	RMSE	RSR	NSE	Reference
AdaBoost	Training	0.9741	0.987	0.2037	0.1324	0.3638	0.162	0.9737	This study
	Testing	0.8791	0.9376	0.4051	0.5918	0.7693	0.3506	0.8771	
CatBoost	Training	0.9618	0.9807	0.3272	0.1988	0.4461	0.1987	0.9605	
	Testing	0.9297	0.9642	0.3845	0.3772	0.6145	0.28	0.9216	
XGBoost	Training	0.9905	0.9952	0.1491	0.0485	0.2203	0.0981	0.9904	
	Testing	0.9251	0.9618	0.3642	0.3723	0.6101	0.278	0.9227	
GPR	Training	0.9402	0.9697	0.3403	–	0.5597	0.248	0.938	Ahmad et al.^28^
	Testing	0.894	0.9455	0.544	–	0.8438	0.387	0.851	
Evolutionary Polynomial Regression (EPR)	Training	0.913	–	0.537	–	1.003	–	–	Rezania et al.^68^
	Testing	0.883	–	0.291	–	1.158	–	–	
Artificial Neural Networks (ANN)	Training	0.875	–	0.702	–	1.074	–	–	
	Testing	0.872	–	0.82	–	1.21	–	–	
MLR	Training	0.868	–	0.81	–	1.24	–	–	
	Testing	0.875	–	0.43	–	1.196	–	–	

“–” not reported in the respective reference.

### Radar plot analysis

**Fig. 11:**
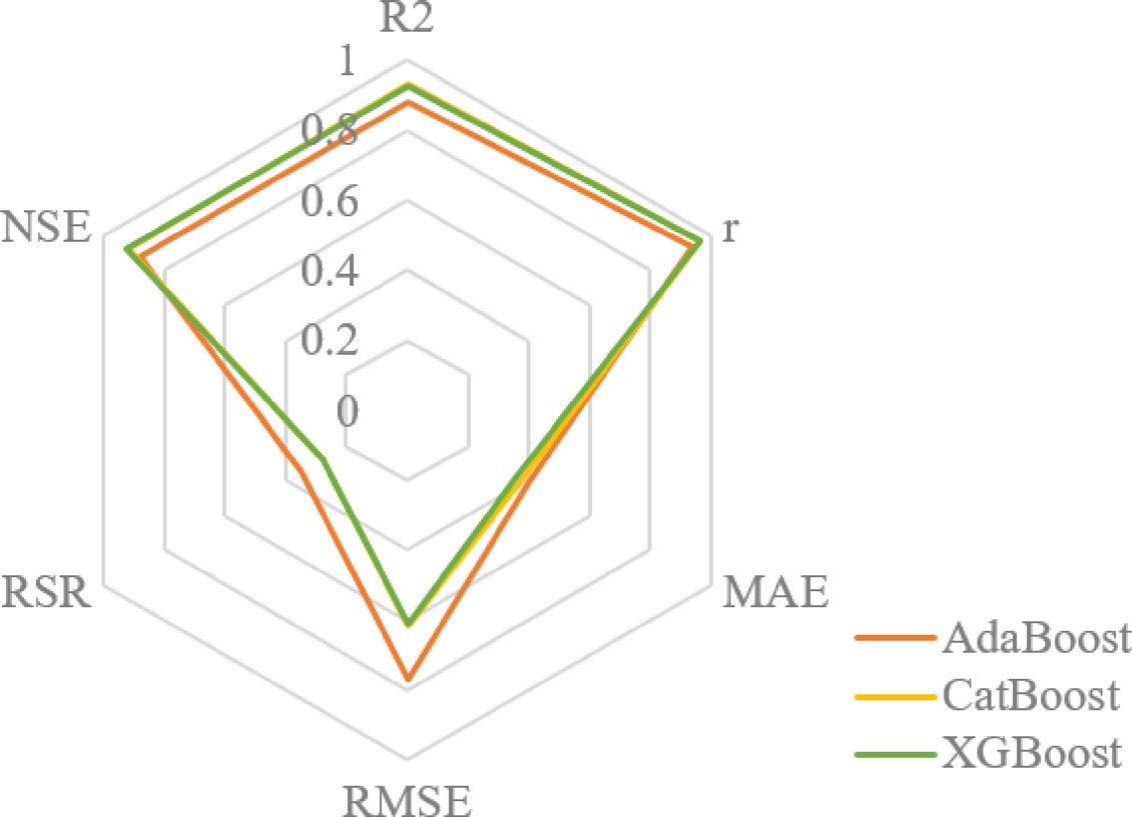
Radar plot shown on a comparison of AdaBoost performance, CatBoost performance and XGBoost performance on the testing phase of six evaluation metrics.

Figure 11 shows a radar chart that graphically demonstrates the relative strengths of each model, which supports the identified trends. The results combined make it clear that XGBoost is the most successful model in the given regression task when tested.

The XGBoost model here proposed is a superior than the current available models in literature since it is more accurate and robust in predictive power but low on computational overhead. It can capture high-dimensional, nonlinear interactions between features using little data preprocessing, leading to more efficient and better generalization of models.

### Rank analysis

To obtain a fair and comprehensive comparison among all the proposed machine learning models, rank analysis was carried out using several metrics (e.g., RMSE, MAE, R^2^, NSE). Models were ranked separately by each metric such that the model with the highest score acquires rank 1, then lower ranks sequentially. The numerical sum of ranks of all metrics was then calculated to obtain the overall position of each model. The statistical performance indicators were analyzed using rank analysis to determine the efficiency of the training and testing models. Table 5 indicates that XGBoost model scored the best in terms of accuracy on testing, with a rank value of 19, followed by CatBoost that had a rank value of 18. In the training phase, XGBoost was ranked the best one being ranked highest with a total rank value of 21 as compared to the other models therefore proving it to be robust and reliable irrespective of the dataset.

**Table 5:** Rank analysis of machine learning models based on statistical performance indicators for training and testing stages.

Dataset	Model	Performance Indicators						*Score*	References
		*R* ^2^	*MAE*	*RMSE*	*R* ^2^	*MAE*	*RMSE*		
Training	AdaBoost	0.9741	0.2037	0.3638	6	6	6	18	Present study
	CatBoost	0.9618	0.3272	0.4461	5	5	5	15	
	XGBoost	0.9905	0.1491	0.2203	7	7	7	21	
	GPR	0.9402	0.3403	0.5597	4	4	4	12	Ahmad et al.^28^
	EPR	0.913	0.537	1.003	3	3	3	9	Rezania et al.^68^
	ANN	0.875	0.702	1.074	2	2	2	6	
	MLR	0.868	0.81	1.24	1	1	1	3	
Testing	AdaBoost	0.8791	0.4051	0.7693	3	4	5	12	Present study
	CatBoost	0.9297	0.3845	0.6145	7	5	6	18	
	XGBoost	0.9251	0.3642	0.6101	6	6	7	19	
	GPR	0.894	0.544	0.8438	5	2	4	11	Ahmad et al.^28^
	EPR	0.883	0.291	1.158	4	7	3	14	Rezania et al.^68^
	ANN	0.872	0.82	1.21	1	1	1	3	
	MLR	0.875	0.43	1.196	2	3	2	7	

### Wilcoxon signed-rank test

The Wilcoxon Signed-Rank Test was used to statistically compare the differences between the three developed models, AdaBoost, CatBoost, and XGBoost. The test was used to compare the predictive performance of the models. The null hypothesis (H₀) states that the median difference between paired observations is zero, indicating no significant difference. A significance level of α = 0.05 was used. The findings demonstrate that AdaBoost and CatBoost differ considerably (*p* = 0.011), as do CatBoost and XGBoost (*p* = 0.010), whereas AdaBoost and XGBoost do not differ significantly (*p* = 0.712). This implies that CatBoost’s predicts diverge more than the others, although AdaBoost and XGBoost are more consistent. The statistical study shows that AdaBoost and XGBoost produce more consistent predictions in comparison to the observed values, as indicated by the non-significant differences. CatBoost, on the other hand, behaves statistically differently, which could reflect model bias or sensitivity to input features. The lack of a substantial difference between AdaBoost and XGBoost indicates that both models perform similarly in capturing the dataset’s underlying patterns. As a result, these models may be considered reliable for prediction purposes in the specific application.

### Sensitivity analysis

The top-ranked XGBoost model (rank value = 21) was used in sensitivity analysis with the Yang and Zhang method as used by Ahmad et al. [69] to determine the individual effect of each input parameter on the prediction of liquefaction-induced lateral displacement and is presented in the Eq. (11):

11 $$\:{r}_{ij}=\frac{\sum\:_{k=1}^{n}({Y}_{ik}\times\:{Y}_{ok})}{\sqrt{\sum\:_{k=1}^{n}{Y}_{ik}^{2}\sum\:_{k=1}^{n}{Y}_{ok}^{2}}}$$

where:r_ij_ correlation coefficient representing the effect of the *i*-th input variable on the *j*-th output parameter. Its value is restricted between 0 and 1; a larger value indicates a stronger influence of the input on the output (here, *D*_*H*_).

i: index of the input variable.

j: index of the output parameter.

o: refers to the observed output values.

k: index of the data point, with *k* = 1, 2, …, *n*.

In this study, *n* = 198 (number of data points), *Y*_ik_ is the value of the *i*-th input for the *k*-th record, and *Y*_ik_ is the corresponding observed output.

**Fig. 12:**
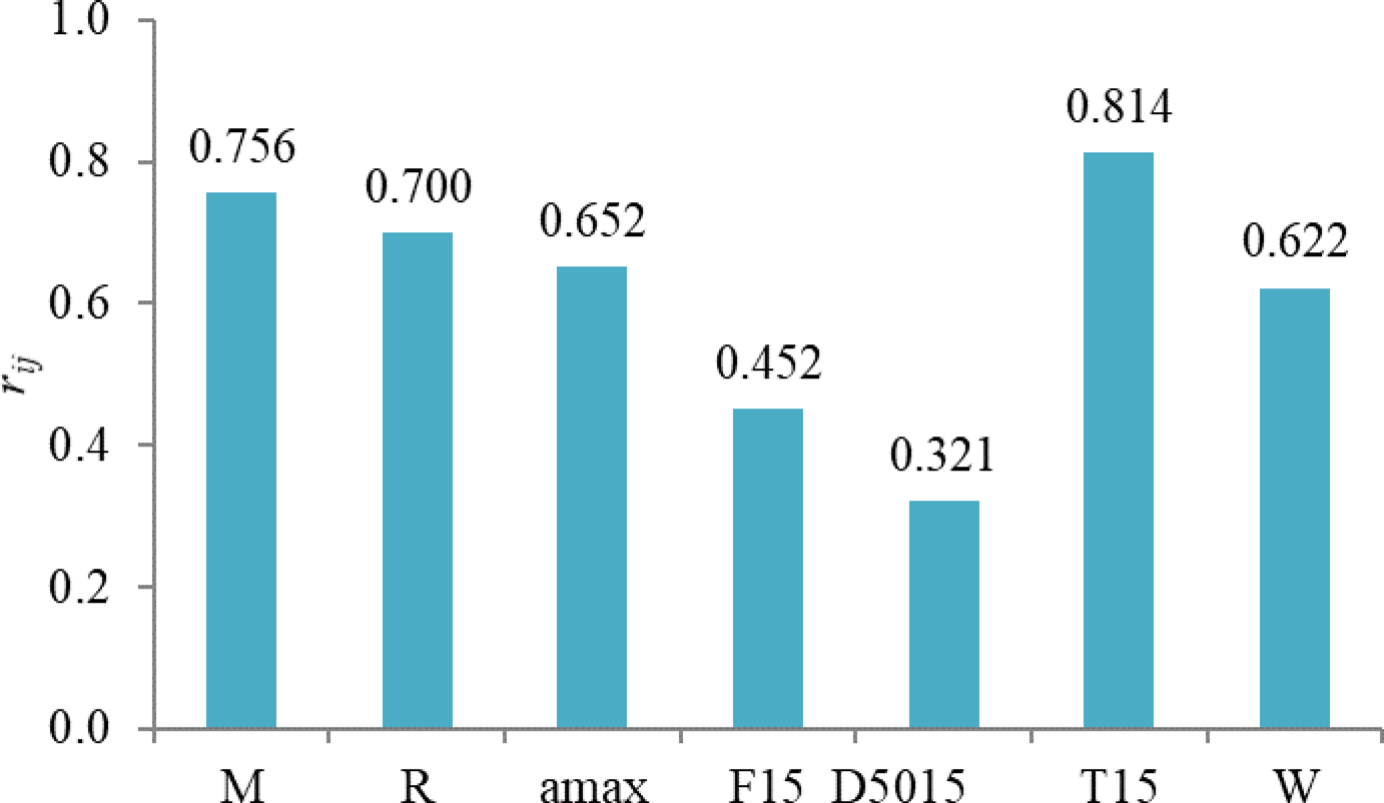
Sensitivity analysis of input parameters.

The *r*_ij_ values reflect the specific input–output sensitivities observed for the XGBoost model used in this study and may differ under alternative modeling approaches. The findings from the Fig. 12 revealed that accumulative thickness of saturated layers with adjusted SPT number SPT number (N_1_)_60_ < 15 (T_15_) was the most sensitive (0.814), followed by earthquake magnitude (M) at 0.756 and horizontal distance to the seismic energy source (R) at 0.700. Peak ground acceleration (*a*_*max*_) and free-face ratio (W) were also found to be highly influential with sensitivities of 0.652 and 0.622 respectively as compared to the average fines content in T_15_ (F_15_) and average particle size in T_15_ (D50_15_) with sensitivities of 0.452 and 0.321 respectively. From a geotechnical perspective, mitigation of the most influential factors may be realized by (i) decreasing T_15_ through densification or replacement of liquefiable layers, drainage or preloading to reduce saturation potential; (ii) mitigating the seismic demand level as a result of high M and *a*_*max*_ by site selection, setback, or methods of ground motion isolation techniques; and (iii) altering geometry or slope profile by decreasing the free-face ratio (W), and hence lowering driving forces of lateral displacement. These confined actions respond to the most sensitive parameters thus minimizing the lateral displacement facilitating the enhanced site resilience to the risks of liquefaction-induced hazards.

### Taylor diagram of model performance

**Fig. 13:**
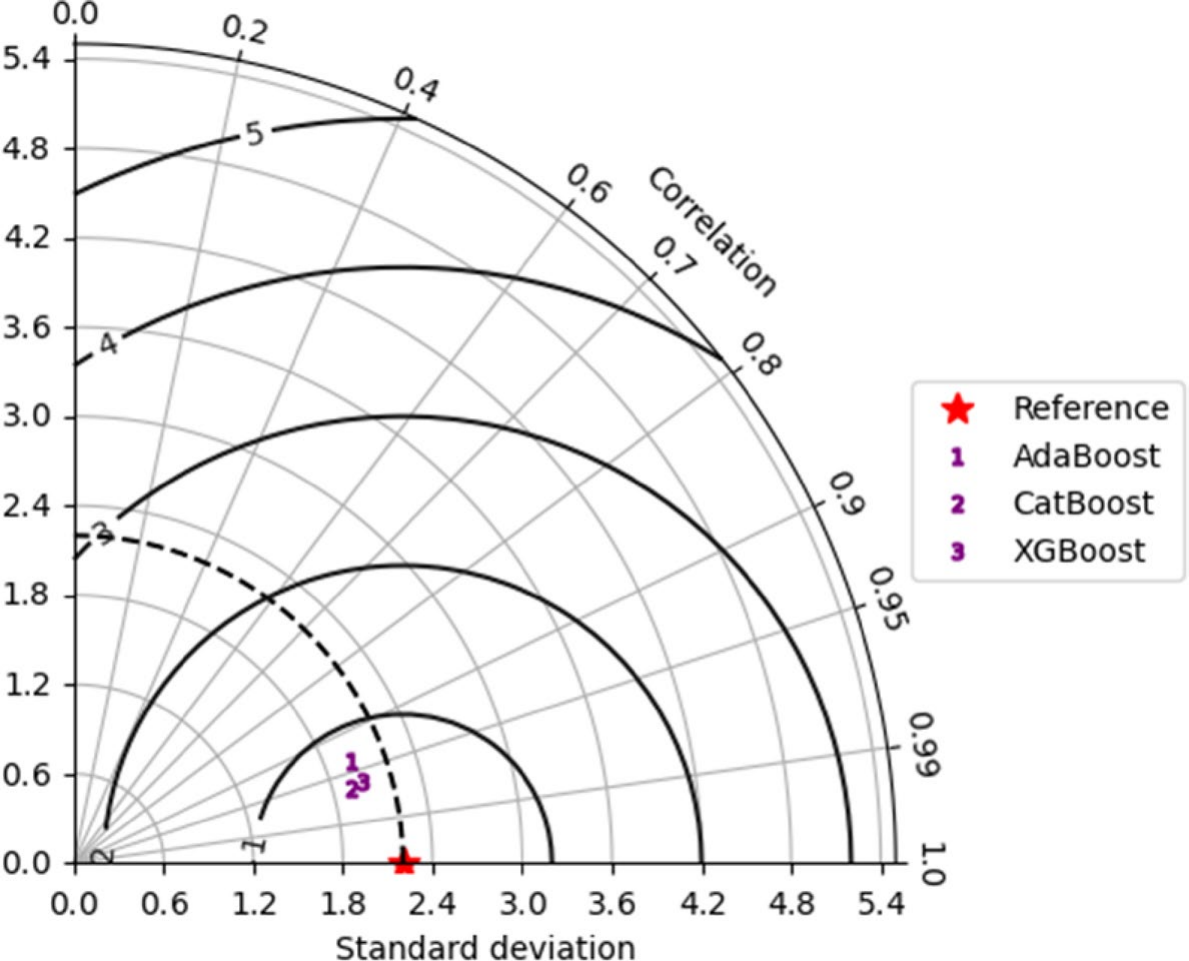
Taylor diagram for model scores by AdaBoost, CatBoost, and XGBoost on liquefaction-induced lateral displacement predictive performance.

The level of agreement can be characterized conveniently in terms of a Taylor diagram (see Fig. 13). The radial distance from the origin here is plotted as standard deviation. The RMS error scales with the difference in standard deviation units of actual and projected fields. The azimuthal angle refers to the correlation coefficient [70]. A Taylor diagram was used to assess the overall performance of the machine learning models in a simultaneous comparison of correlation coefficient, standard deviation and root mean square error to the observed data. This graphical method gives a combined evaluation, with a model that is nearer to the reference point showing more precision and consistency.

The proposed AdaBoost, CatBoost and XGBoost models can predict the liquefaction induced lateral displacement with a high level of accuracy. In addition, the expected accuracy of the model is largely determined by values of parameters used in training and affects by distribution of variables.

## Conclusions

The three models were built for the prediction of liquefaction-induced lateral displacement using AdaBoost, CatBoost and XGBoost algorithms. The models were developed based on seven input parameters and one dependent parameter, *D*_*H*_. Statistical indexes including R^2^, *r*, MAE, RMSE, RSR and NSE were applied to evaluate the models. The study dataset included 247 cases of which 198 were used for training and 49 were used to evaluate each model. The XGBoost has R^2^ =0.9905, MAE = 0.1491, MSE = 0.0485, RMSE = 0.2203 in the training phase while for testing phase R^2^ = 0.9251, model *r* = 0.9618, MAE = 0.3642, MSE = 0.3723, RMSE = 0.6101. For the training phase CatBoost has R^2^ = 0.9618, *r* = 0.9807, MAE = 0.3272, MSE = 0.1988, RMSE = 0.4461, whereas for the testing phase R^2^ = 0.9297, *r* = 0.9642, MAE = 0.3845, MSE = 0.3772, RMSE = 0.6145. AdaBoost has R^2^ = 0.9741, *r* = 0.987, MAE = 0.2037, MSE = 0.1324, RMSE = 0.3638 for the training phase and R^2^ = 0.8791, *r* = 0.9376, MAE = 0.4501, MSE = 0.5918, RMSE = 0.7693 for the testing phase. Based on the comparison, it can be concluded that XGBoost and CatBoost give better performances in comparison to AdaBoost, GPR, EPR ANN and MLR. The sensitivity analysis showed that the values of parameters T_15_ (*r*_*ij*_=0.814), M (*r*_*ij*_=0.756) and R (*r*_*ij*_=0.700) were important for the output parameter. The *r*_*ij*_ value of D50_15_ is the lowest (0.321) compared with other seven parameters, and the parameter T_15_ has the most influence on the output parameter, *D*_*H*_. The study is confined to the free-face liquefaction scenario, training and validation of the models was carried out within the ranges of compiled geotechnical, seismic, and topographic parameters and is therefore not extrapolate to other site conditions or extreme loading. The data-driven model does not have constitutive behavior of soil embedded in it, which weakens physical interpretability. Future research should extend the data range to other sites and seismic conditions and implement physics-informed or hybrid ML solutions to combine empirical learning and the geomechanical framework to achieve better levels of robustness and generalization.

## Supplementary Information

Below is the link to the electronic supplementary material.

Supplementary Material 1

## Data Availability

All data supporting the findings of this study are available within the paper and its Supplementary Information.
