# Supplementary material for: Performance evaluation of machine learning algorithms for predicting liquefaction-induced lateral displacement
Source: Sci Rep. 2026 Apr 30;16:20188. doi: 10.1038/s41598-026-50670-4 (PMC13324425; doi:10.1038/s41598-026-50670-4)
Supplement: Supplementary file 1 — Supplementary Material 1 [file 41598_2026_50670_MOESM1_ESM.docx]

Table S1: Lateral displacement case history data collected from Youd et al. [47], Cetin et al. [48], and Chu et al. [49].

| Earthquake | *M* | *R* (km) | *a_max_* (g) | *F_15_* (%) | *D50_15_* (mm) | *T_15_* (m) | *W* (%) | *D_H_* (m) |
| --- | --- | --- | --- | --- | --- | --- | --- | --- |
| 1989, Loma Prieta | 7 | 27.2 | 0.2 | 1 | 0.6 | 3.4 | 29.73 | 0.26 |
| 1971, San Fernando | 6.4 | 0.5 | 0.68 | 47 | 0.08 | 5.3 | 19.96 | 2.93 |
| 1964, Niigata | 7.5 | 21 | 0.32 | 5 | 0.35 | 12.7 | 3.06 | 1.01 |
| 1979, Imperial Valley | 6.5 | 2 | 0.49 | 20 | 0.12 | 3 | 8.57 | 2.63 |
| 1995, Hyogo-Ken Nanbu | 6.8 | 7.5 | 0.35 | 12.6 | 0.47 | 14.2 | 13.95 | 1.18 |
| 1995, Hyogo-Ken Nanbu | 6.8 | 6 | 0.38 | 13.4 | 0.94 | 12.5 | 9.25 | 1.01 |
| 1964, Niigata | 7.5 | 21 | 0.32 | 4 | 0.34 | 13.6 | 3.15 | 5.2 |
| 1979, Imperial Valley | 6.5 | 2 | 0.49 | 32 | 0.09 | 1.5 | 6.25 | 0.37 |
| 1964, Niigata | 7.5 | 21 | 0.32 | 24 | 0.19 | 8.6 | 5.36 | 0.82 |
| 1964, Niigata | 7.5 | 21 | 0.32 | 6 | 0.35 | 0.5 | 3.43 | 1.1 |
| 1979, Imperial Valley | 6.5 | 2 | 0.49 | 23 | 0.11 | 2 | 7.89 | 2.04 |
| 1964, Niigata | 7.5 | 21 | 0.32 | 32 | 0.1 | 2.4 | 2.03 | 0.54 |
| 1964, Niigata | 7.5 | 21 | 0.32 | 26 | 0.16 | 2.5 | 20.61 | 0.91 |
| 1964, Niigata | 7.5 | 21 | 0.32 | 6 | 0.35 | 0.5 | 22.37 | 0.88 |
| 1964, Niigata | 7.5 | 21 | 0.32 | 10 | 0.25 | 11.3 | 29.7 | 5.03 |
| 1979, Imperial Valley | 6.5 | 2 | 0.49 | 17 | 0.12 | 3.6 | 3.08 | 0.92 |
| 1964, Niigata | 7.5 | 21 | 0.32 | 6 | 0.29 | 7.5 | 7.32 | 3.75 |
| 1971, San Fernando | 6.4 | 0.5 | 0.68 | 47 | 0.08 | 5.6 | 4.7 | 0.47 |
| 1979, Imperial Valley | 6.5 | 2 | 0.49 | 15 | 0.12 | 3.8 | 6.56 | 2.02 |
| 1971, San Fernando | 6.4 | 0.5 | 0.68 | 47 | 0.08 | 6.5 | 5.08 | 0.52 |
| 1964, Niigata | 7.5 | 21 | 0.32 | 24 | 0.19 | 8.6 | 8.78 | 0.93 |
| 1979, Imperial Valley | 6.6 | 6 | 0.36 | 70 | 0.04 | 0.2 | 4.26 | 0.01 |
| 1964, Niigata | 7.5 | 21 | 0.32 | 12 | 0.27 | 12.2 | 5.01 | 2.36 |
| 1995, Hyogo-Ken Nanbu | 6.8 | 8 | 0.34 | 14.6 | 1.98 | 16 | 6.67 | 0.45 |
| 1995, Hyogo-Ken Nanbu | 6.8 | 8 | 0.34 | 14.6 | 1.98 | 16 | 16.82 | 0.93 |
| 1964, Niigata | 7.5 | 21 | 0.32 | 24 | 0.19 | 8.6 | 24.02 | 3.07 |
| 1964, Niigata | 7.5 | 21 | 0.32 | 9 | 0.26 | 11.3 | 19.62 | 10.16 |
| 1971, San Fernando | 6.4 | 0.5 | 0.68 | 47 | 0.08 | 4.6 | 20.3 | 3.16 |
| 1995, Hyogo-Ken Nanbu | 6.8 | 7.5 | 0.35 | 12.6 | 0.47 | 14.2 | 10.4 | 0.89 |
| 1979, Imperial Valley | 6.6 | 6 | 0.36 | 54 | 0.12 | 1.8 | 10.66 | 0.01 |
| 1971, San Fernando | 6.4 | 0.5 | 0.68 | 47 | 0.08 | 3.6 | 20.34 | 3.18 |
| 1964, Niigata | 7.5 | 21 | 0.32 | 12 | 0.26 | 12.3 | 5.76 | 1.49 |
| 1964, Niigata | 7.5 | 21 | 0.32 | 31 | 0.12 | 2.4 | 3.26 | 1.25 |
| 1964, Niigata | 7.5 | 21 | 0.32 | 10 | 0.39 | 9 | 3.27 | 2.48 |
| 1964, Niigata | 7.5 | 21 | 0.32 | 32 | 0.11 | 2.4 | 2.09 | 1.32 |
| 1964, Niigata | 7.5 | 21 | 0.32 | 14 | 0.36 | 7.1 | 19.62 | 3.34 |
| 1964, Niigata | 7.5 | 21 | 0.32 | 4 | 0.57 | 8.6 | 2.82 | 1.23 |
| 1979, Imperial Valley | 6.5 | 2 | 0.49 | 17 | 0.12 | 3.7 | 9.6 | 4 |
| 1971, San Fernando | 6.4 | 0.5 | 0.68 | 47 | 0.08 | 3 | 17.07 | 1.81 |
| 1971, San Fernando | 6.4 | 0.5 | 0.68 | 47 | 0.08 | 2.3 | 13.59 | 2.14 |
| 1964, Niigata | 7.5 | 21 | 0.32 | 11 | 0.26 | 11.9 | 5.93 | 2.97 |
| 1964, Niigata | 7.5 | 21 | 0.32 | 5 | 0.32 | 15.6 | 4.94 | 7.36 |
| 1964, Niigata | 7.5 | 21 | 0.32 | 16 | 0.22 | 9.6 | 3.06 | 2.41 |
| 1979, Imperial Valley | 6.5 | 2 | 0.49 | 22 | 0.11 | 2.6 | 3.68 | 0.31 |
| 1964, Niigata | 7.5 | 21 | 0.32 | 24 | 0.19 | 8.6 | 18.49 | 1.78 |
| 1979, Imperial Valley | 6.5 | 2 | 0.49 | 23 | 0.11 | 2.4 | 6.35 | 1.41 |
| 1964, Niigata | 7.5 | 21 | 0.32 | 11 | 0.27 | 12 | 4.83 | 1.84 |
| 1964, Niigata | 7.5 | 21 | 0.32 | 6 | 0.32 | 12.4 | 4.82 | 3.66 |
| 1964, Niigata | 7.5 | 21 | 0.32 | 12 | 0.26 | 12.4 | 5.01 | 1.75 |
| 1906, San Francisco | 7.9 | 27 | 0.24 | 23 | 0.25 | 7.2 | 22.02 | 1.84 |
| 1971, San Fernando | 6.4 | 0.5 | 0.68 | 47 | 0.08 | 1.6 | 20.41 | 2.45 |
| 1964, Niigata | 7.5 | 21 | 0.32 | 31 | 0.12 | 2.4 | 3.35 | 0.69 |
| 1971, San Fernando | 6.4 | 0.5 | 0.68 | 47 | 0.08 | 4.8 | 19.61 | 2.78 |
| 1964, Niigata | 7.5 | 21 | 0.32 | 7 | 0.35 | 9.8 | 4.5 | 0.53 |
| 1964, Niigata | 7.5 | 21 | 0.32 | 5 | 0.32 | 15.6 | 7.86 | 8.37 |
| 1964, Niigata | 7.5 | 21 | 0.32 | 5 | 0.32 | 13.9 | 5.77 | 4.58 |
| 1995, Hyogo-Ken Nanbu | 6.8 | 5.5 | 0.39 | 10 | 1.36 | 15 | 14.56 | 1.34 |
| 1995, Hyogo-Ken Nanbu | 6.8 | 5.5 | 0.39 | 10 | 1.36 | 15 | 30.21 | 2.83 |
| 1979, Imperial Valley | 6.5 | 2 | 0.49 | 23 | 0.11 | 2 | 6.15 | 1.1 |
| 1964, Niigata | 7.5 | 21 | 0.32 | 12 | 0.26 | 12 | 9.18 | 4.4 |
| 1964, Niigata | 7.5 | 21 | 0.32 | 12 | 0.24 | 11.8 | 5.54 | 4 |
| 1964, Niigata | 7.5 | 21 | 0.32 | 12 | 0.26 | 12.2 | 5.36 | 2.38 |
| 1964, Niigata | 7.5 | 21 | 0.32 | 5 | 0.44 | 10.1 | 2.42 | 1.25 |
| 1979, Imperial Valley | 6.5 | 2 | 0.49 | 22 | 0.11 | 2.7 | 6.45 | 1.53 |
| 1964, Niigata | 7.5 | 21 | 0.32 | 11 | 0.28 | 12.1 | 3.68 | 2.09 |
| 1964, Niigata | 7.5 | 21 | 0.32 | 6 | 0.35 | 0.5 | 3.39 | 0.86 |
| 1964, Niigata | 7.5 | 21 | 0.32 | 14 | 0.25 | 12.6 | 13.73 | 6.27 |
| 1964, Niigata | 7.5 | 21 | 0.32 | 5 | 0.4 | 7.9 | 3.59 | 1.46 |
| 1964, Niigata | 7.5 | 21 | 0.32 | 5 | 0.32 | 15.6 | 17.75 | 9.15 |
| 1964, Niigata | 7.5 | 21 | 0.32 | 6 | 0.35 | 0.5 | 4.26 | 0.72 |
| 1964, Niigata | 7.5 | 21 | 0.32 | 6 | 0.29 | 14.3 | 6.51 | 3.61 |
| 1971, San Fernando | 6.4 | 0.5 | 0.68 | 47 | 0.08 | 2.7 | 15.43 | 2.02 |
| 1964, Niigata | 7.5 | 21 | 0.32 | 8 | 0.23 | 6.8 | 1.85 | 0.91 |
| 1995, Hyogo-Ken Nanbu | 6.8 | 6.5 | 0.37 | 10 | 1.88 | 12.5 | 5.16 | 0.34 |
| 1971, San Fernando | 6.4 | 0.5 | 0.68 | 47 | 0.08 | 2 | 13.59 | 1.46 |
| 1964, Niigata | 7.5 | 21 | 0.32 | 24 | 0.19 | 8.6 | 5.29 | 1.64 |
| 1964, Niigata | 7.5 | 21 | 0.32 | 5 | 0.36 | 13.6 | 8.52 | 4.77 |
| 1979, Imperial Valley | 6.5 | 2 | 0.49 | 23 | 0.11 | 2.9 | 7.02 | 1.43 |
| 1964, Niigata | 7.5 | 21 | 0.32 | 5 | 0.5 | 10.9 | 4.77 | 0.81 |
| 1964, Niigata | 7.5 | 21 | 0.32 | 5 | 0.35 | 12.7 | 9.12 | 6 |
| 1995, Hyogo-Ken Nanbu | 6.8 | 5.5 | 0.39 | 10 | 1.36 | 15 | 56.8 | 2.48 |
| 1979, Imperial Valley | 6.5 | 2 | 0.49 | 25 | 0.1 | 2.5 | 6.78 | 0.72 |
| 1964, Niigata | 7.5 | 21 | 0.32 | 15 | 0.25 | 9.6 | 2.68 | 1.89 |
| 1964, Niigata | 7.5 | 21 | 0.32 | 24 | 0.19 | 8.6 | 8.19 | 2.2 |
| 1971, San Fernando | 6.4 | 0.5 | 0.68 | 47 | 0.08 | 4 | 18.87 | 3.26 |
| 1964, Niigata | 7.5 | 21 | 0.32 | 3 | 0.35 | 13.3 | 4.05 | 4.76 |
| 1964, Alaska | 9.2 | 60 | 0.3 | 21 | 1.35 | 3.4 | 24.59 | 1.86 |
| 1979, Imperial Valley | 6.5 | 2 | 0.49 | 15 | 0.12 | 4 | 6.56 | 1.48 |
| 1964, Niigata | 7.5 | 21 | 0.32 | 11 | 0.26 | 12 | 6.53 | 2.51 |
| 1964, Niigata | 7.5 | 21 | 0.32 | 5 | 0.32 | 15.6 | 17.75 | 9.49 |
| 1964, Niigata | 7.5 | 21 | 0.32 | 11 | 0.24 | 11.6 | 11.06 | 8.19 |
| 1971, San Fernando | 6.4 | 0.5 | 0.68 | 47 | 0.08 | 2.7 | 20.47 | 3.16 |
| 1964, Niigata | 7.5 | 21 | 0.32 | 13 | 0.29 | 13.6 | 2.76 | 1.01 |
| 1964, Niigata | 7.5 | 21 | 0.32 | 7 | 0.34 | 10.5 | 6.03 | 5.43 |
| 1979, Imperial Valley | 6.5 | 2 | 0.49 | 17 | 0.12 | 3.7 | 6.78 | 2.3 |
| 1979, Imperial Valley | 6.5 | 2 | 0.49 | 21 | 0.11 | 1.6 | 4.8 | 0.67 |
| 1964, Niigata | 7.5 | 21 | 0.32 | 6 | 0.45 | 10.5 | 5.84 | 1.86 |
| 1964, Niigata | 7.5 | 21 | 0.32 | 5 | 0.32 | 15.5 | 9.98 | 6.02 |
| 1964, Niigata | 7.5 | 21 | 0.32 | 6 | 0.39 | 9.2 | 4.87 | 1.86 |
| 1964, Niigata | 7.5 | 21 | 0.32 | 12 | 0.24 | 11.9 | 5.06 | 3.98 |
| 1971, San Fernando | 6.4 | 0.5 | 0.68 | 47 | 0.08 | 5.9 | 4.89 | 0.54 |
| 1964, Niigata | 7.5 | 21 | 0.32 | 5 | 0.32 | 15.6 | 17.05 | 9.29 |
| 1964, Niigata | 7.5 | 21 | 0.32 | 15 | 0.32 | 11.3 | 2.86 | 1.41 |
| 1964, Niigata | 7.5 | 21 | 0.32 | 16 | 0.31 | 11 | 3.06 | 1.3 |
| 1964, Niigata | 7.5 | 21 | 0.32 | 18 | 0.21 | 6.7 | 4.45 | 0.9 |
| 1906, San Francisco | 7.9 | 24 | 0.26 | 30 | 0.16 | 1.5 | 17.76 | 0.92 |
| 1964, Niigata | 7.5 | 21 | 0.32 | 15 | 0.32 | 7 | 7.72 | 1.92 |
| 1964, Niigata | 7.5 | 21 | 0.32 | 11 | 0.28 | 12.1 | 2.88 | 1.56 |
| 1964, Niigata | 7.5 | 21 | 0.32 | 6 | 0.38 | 11.6 | 3.22 | 2.71 |
| 1964, Niigata | 7.5 | 21 | 0.32 | 5 | 0.32 | 15.6 | 5.25 | 7.19 |
| 1964, Niigata | 7.5 | 21 | 0.32 | 16 | 0.3 | 10.8 | 3.68 | 0.71 |
| 1964, Niigata | 7.5 | 21 | 0.32 | 13 | 0.38 | 7.2 | 20.55 | 3.28 |
| 1964, Niigata | 7.5 | 21 | 0.32 | 9 | 0.4 | 13 | 2.05 | 1.11 |
| 1964, Niigata | 7.5 | 21 | 0.32 | 13 | 0.25 | 12.5 | 16.07 | 7.4 |
| 1995, Hyogo-Ken Nanbu | 6.8 | 8 | 0.34 | 14.6 | 1.98 | 16 | 18 | 0.97 |
| 1964, Niigata | 7.5 | 21 | 0.32 | 6 | 0.37 | 12.7 | 7.05 | 3.54 |
| 1979, Imperial Valley | 6.5 | 2 | 0.49 | 25 | 0.1 | 2.5 | 9.84 | 2.63 |
| 1964, Niigata | 7.5 | 21 | 0.32 | 6 | 0.35 | 0.5 | 2.68 | 0.82 |
| 1964, Niigata | 7.5 | 21 | 0.32 | 9 | 0.39 | 9.3 | 3.72 | 1.96 |
| 1995, Hyogo-Ken Nanbu | 6.8 | 8 | 0.34 | 14.6 | 1.98 | 16 | 20.69 | 0.9 |
| 1964, Niigata | 7.5 | 21 | 0.32 | 5 | 0.39 | 7.3 | 2.76 | 1.23 |
| 1964, Niigata | 7.5 | 21 | 0.32 | 24 | 0.19 | 8.6 | 12.86 | 2.74 |
| 1964, Niigata | 7.5 | 21 | 0.32 | 12 | 0.25 | 12.1 | 16.72 | 4.88 |
| 1964, Niigata | 7.5 | 21 | 0.32 | 11 | 0.27 | 12.1 | 3.38 | 1.83 |
| 1964, Niigata | 7.5 | 21 | 0.32 | 5 | 0.32 | 15.6 | 5.77 | 7.21 |
| 1979, Imperial Valley | 6.5 | 2 | 0.49 | 22 | 0.11 | 1.8 | 6.67 | 1.13 |
| 1964, Niigata | 7.5 | 21 | 0.32 | 2 | 0.33 | 10.4 | 8.89 | 4.76 |
| 1964, Niigata | 7.5 | 21 | 0.32 | 13 | 0.25 | 12.4 | 35 | 7.67 |
| 1995, Hyogo-Ken Nanbu | 6.8 | 7.5 | 0.35 | 12.6 | 0.47 | 14.2 | 18.56 | 1.33 |
| 1964, Alaska | 9.2 | 100 | 0.2 | 13 | 1 | 10.4 | 7.03 | 1.38 |
| 1964, Niigata | 7.5 | 21 | 0.32 | 7 | 0.32 | 9.4 | 2.99 | 1.31 |
| 1964, Niigata | 7.5 | 21 | 0.32 | 4 | 0.34 | 13.5 | 3.36 | 3.46 |
| 1987, Superstition Hills | 6.6 | 23 | 0.15 | 27 | 0.09 | 3.5 | 17.91 | 0.19 |
| 1964, Niigata | 7.5 | 21 | 0.32 | 11 | 0.26 | 11.6 | 11.32 | 3.78 |
| 1964, Niigata | 7.5 | 21 | 0.32 | 3 | 0.44 | 11.3 | 3.82 | 1.52 |
| 1964, Niigata | 7.5 | 21 | 0.32 | 13 | 0.29 | 13 | 3.1 | 0.56 |
| 1964, Niigata | 7.5 | 21 | 0.32 | 28 | 0.14 | 2.5 | 4.79 | 0.88 |
| 1964, Niigata | 7.5 | 21 | 0.32 | 5 | 0.32 | 15.6 | 19.62 | 7.7 |
| 1971, San Fernando | 6.4 | 0.5 | 0.68 | 47 | 0.08 | 4.5 | 19.26 | 1.99 |
| 1964, Niigata | 7.5 | 21 | 0.32 | 3 | 0.44 | 11.3 | 4.87 | 1.9 |
| 1964, Niigata | 7.5 | 21 | 0.32 | 9 | 0.37 | 10 | 16.4 | 6.5 |
| 1995, Hyogo-Ken Nanbu | 6.8 | 8 | 0.34 | 14.6 | 1.98 | 16 | 14.63 | 0.66 |
| 1964, Niigata | 7.5 | 21 | 0.32 | 7 | 0.35 | 9.8 | 3.9 | 2.87 |
| 1971, San Fernando | 6.4 | 0.5 | 0.68 | 47 | 0.08 | 1 | 20.27 | 1 |
| 1964, Niigata | 7.5 | 21 | 0.32 | 12 | 0.25 | 12.2 | 12.47 | 4.83 |
| 1987, Superstition Hills | 6.6 | 23 | 0.15 | 22 | 0.09 | 3.3 | 41.38 | 0.21 |
| 1979, Imperial Valley | 6.5 | 2 | 0.49 | 30 | 0.09 | 1.8 | 8.05 | 1.03 |
| 1979, Imperial Valley | 6.5 | 2 | 0.49 | 22 | 0.11 | 2.7 | 8.05 | 2.12 |
| 1964, Niigata | 7.5 | 21 | 0.32 | 5 | 0.34 | 13.8 | 12.01 | 8.73 |
| 1964, Niigata | 7.5 | 21 | 0.32 | 24 | 0.19 | 8.6 | 25.93 | 3.57 |
| 1995, Hyogo-Ken Nanbu | 6.8 | 6.5 | 0.37 | 10 | 1.88 | 12.5 | 9.84 | 1.03 |
| 1964, Niigata | 7.5 | 21 | 0.32 | 14 | 0.25 | 12.6 | 11.32 | 3.51 |
| 1964, Niigata | 7.5 | 21 | 0.32 | 17 | 0.24 | 6.8 | 4.26 | 1.37 |
| 1964, Alaska | 9.2 | 60 | 0.3 | 23 | 1.47 | 3.8 | 16.07 | 1.58 |
| 1964, Niigata | 7.5 | 21 | 0.32 | 11 | 0.25 | 11.6 | 17.05 | 8.29 |
| 1971, San Fernando | 6.4 | 0.5 | 0.68 | 47 | 0.08 | 3.1 | 18.26 | 2.04 |
| 1964, Niigata | 7.5 | 21 | 0.32 | 5 | 0.31 | 14.1 | 16.4 | 8.52 |
| 1964, Niigata | 7.5 | 21 | 0.32 | 24 | 0.19 | 8.6 | 5.76 | 1.27 |
| 1964, Niigata | 7.5 | 21 | 0.32 | 15 | 0.25 | 9.5 | 3.04 | 2.68 |
| 1964, Niigata | 7.5 | 21 | 0.32 | 13 | 0.27 | 11.8 | 2.27 | 1.56 |
| 1964, Niigata | 7.5 | 21 | 0.32 | 13 | 0.25 | 12.4 | 12.47 | 3.21 |
| 1979, Imperial Valley | 6.5 | 2 | 0.49 | 25 | 0.11 | 2.2 | 3.68 | 0.47 |
| 1964, Niigata | 7.5 | 21 | 0.32 | 24 | 0.19 | 8.6 | 7.14 | 2.15 |
| 1964, Niigata | 7.5 | 21 | 0.32 | 24 | 0.19 | 8.6 | 5.15 | 1.06 |
| 1964, Niigata | 7.5 | 21 | 0.32 | 7 | 0.43 | 10.4 | 15.25 | 2.25 |
| 1964, Niigata | 7.5 | 21 | 0.32 | 5 | 0.35 | 16.7 | 11.06 | 2.91 |
| 1964, Niigata | 7.5 | 21 | 0.32 | 8 | 0.15 | 3.7 | 1.64 | 0.62 |
| 1987, Superstition Hills | 6.6 | 23 | 0.15 | 43 | 0.07 | 1.7 | 17.52 | 0.11 |
| 1979, Imperial Valley | 6.5 | 2 | 0.49 | 16 | 0.12 | 3.8 | 9.37 | 4.25 |
| 1964, Niigata | 7.5 | 21 | 0.32 | 5 | 0.32 | 15.6 | 7.72 | 7.31 |
| 1999, Chi-Chi | 7.6 | 5 | 0.67 | 20.8 | 0.11 | 0.5 | 7.4 | 0 |
| 1999, Chi-Chi | 7.6 | 5 | 0.67 | 20.8 | 0.11 | 0.8 | 13.7 | 0.45 |
| 1999, Chi-Chi | 7.6 | 5 | 0.67 | 20.8 | 0.11 | 0.8 | 18.4 | 0.55 |
| 1999, Chi-Chi | 7.6 | 5 | 0.67 | 20.8 | 0.11 | 0.8 | 25.2 | 0.8 |
| 1999, Chi-Chi | 7.6 | 5 | 0.67 | 20.8 | 0.11 | 0.8 | 37.3 | 1.05 |
| 1999, Chi-Chi | 7.6 | 5 | 0.67 | 20.8 | 0.11 | 0.8 | 49.9 | 2.05 |
| 1979, Imperial Valley | 6.5 | 2 | 0.49 | 22 | 0.11 | 3 | 10.08 | 3.21 |
| 1964, Niigata | 7.5 | 21 | 0.32 | 13 | 0.25 | 12.5 | 55.68 | 7.13 |
| 1964, Niigata | 7.5 | 21 | 0.32 | 6 | 0.31 | 15.2 | 12.44 | 6.3 |
| 1964, Niigata | 7.5 | 21 | 0.32 | 13 | 0.35 | 11.9 | 2.86 | 1.11 |
| 1964, Niigata | 7.5 | 21 | 0.32 | 11 | 0.25 | 11.6 | 19.37 | 4.28 |
| 1999, Chi-Chi | 7.6 | 5 | 0.67 | 13 | 0.18 | 0.75 | 21.2 | 0.49 |
| 1999, Chi-Chi | 7.6 | 5 | 0.67 | 20.8 | 0.11 | 1.1 | 11.9 | 0 |
| 1999, Chi-Chi | 7.6 | 5 | 0.67 | 20.8 | 0.11 | 1.1 | 26.3 | 0 |
| 1999, Chi-Chi | 7.6 | 5 | 0.67 | 30 | 0.13 | 0.45 | 12.2 | 0.4 |
| 1999, Chi-Chi | 7.6 | 5 | 0.67 | 30 | 0.13 | 0.45 | 14.3 | 0.65 |
| 1999, Chi-Chi | 7.6 | 5 | 0.67 | 30 | 0.13 | 0.45 | 24.6 | 1 |
| 1999, Chi-Chi | 7.6 | 5 | 0.67 | 30 | 0.13 | 0.45 | 57.7 | 1.24 |
| 1999, Chi-Chi | 7.6 | 5 | 0.67 | 31.4 | 0.1 | 1 | 8 | 0.35 |
| 1999, Chi-Chi | 7.6 | 5 | 0.67 | 31.4 | 0.1 | 1 | 10.5 | 0.61 |
| 1999, Chi-Chi | 7.6 | 5 | 0.67 | 31.4 | 0.1 | 1 | 19 | 0.96 |
| 1999, Chi-Chi | 7.6 | 5 | 0.67 | 31.4 | 0.1 | 1 | 31.3 | 2.96 |
| 1999, Chi-Chi | 7.6 | 5 | 0.67 | 48.5 | 0.1 | 1.8 | 9.6 | 0.35 |
| 1999, Chi-Chi | 7.6 | 5 | 0.67 | 48.5 | 0.1 | 1.8 | 11.7 | 0.52 |
| 1999, Chi-Chi | 7.6 | 5 | 0.67 | 48.5 | 0.1 | 1.8 | 13.3 | 0.62 |
| 1999, Chi-Chi | 7.6 | 5 | 0.67 | 48.5 | 0.1 | 1.8 | 23.7 | 1.62 |
| 1999, Kocaeli | 7.4 | 0.5 | 0.57 | 11 | 7.7 | 1.2 | 8 | 0.9 |
| 1987, Superstition Hills | 6.6 | 23 | 0.15 | 44 | 0.07 | 3.6 | 7.5 | 0.01 |
| 1999, Kocaeli | 7.4 | 0.5 | 0.57 | 31 | 0.55 | 1.7 | 6 | 0.1 |
| 1987, Superstition Hills | 6.6 | 23 | 0.15 | 38 | 0.08 | 2.7 | 13.11 | 0.11 |
| 1964, Niigata | 7.5 | 21 | 0.32 | 10 | 0.28 | 12.1 | 3.09 | 1.66 |
| 1964, Niigata | 7.5 | 21 | 0.32 | 5 | 0.36 | 9.6 | 3.72 | 3.26 |
| 1964, Niigata | 7.5 | 21 | 0.32 | 13 | 0.25 | 12.3 | 16.07 | 7.06 |
| 1964, Niigata | 7.5 | 21 | 0.32 | 2 | 0.33 | 10.4 | 13.65 | 5.35 |
| 1995, Hyogo-Ken Nanbu | 6.8 | 5.5 | 0.39 | 10 | 1.36 | 15 | 14.34 | 1.31 |
| 1964, Alaska | 9.2 | 60 | 0.3 | 66 | 0.07 | 3.1 | 48.98 | 1.92 |
| 1964, Niigata | 7.5 | 21 | 0.32 | 13 | 0.26 | 12.6 | 6.23 | 1.87 |
| 1979, Imperial Valley | 6.5 | 2 | 0.49 | 16 | 0.12 | 3.7 | 3.72 | 1.23 |
| 1964, Niigata | 7.5 | 21 | 0.32 | 3 | 0.44 | 11.3 | 3.27 | 0.96 |
| 1964, Niigata | 7.5 | 21 | 0.32 | 7 | 0.33 | 10.6 | 12.01 | 7.95 |
| 1964, Niigata | 7.5 | 21 | 0.32 | 3 | 0.44 | 11.3 | 5.12 | 1.36 |
| 1964, Niigata | 7.5 | 21 | 0.32 | 30 | 0.13 | 2.4 | 4.18 | 0.68 |
| 1964, Niigata | 7.5 | 21 | 0.32 | 4 | 0.34 | 13.6 | 2.99 | 4.85 |
| 1964, Niigata | 7.5 | 21 | 0.32 | 28 | 0.14 | 2.5 | 20.61 | 1.06 |
| 1979, Imperial Valley | 6.5 | 2 | 0.49 | 18 | 0.12 | 3.4 | 9.16 | 3.82 |
| 1964, Niigata | 7.5 | 21 | 0.32 | 11 | 0.28 | 12.1 | 3.86 | 1.93 |
| 1964, Niigata | 7.5 | 21 | 0.32 | 2 | 0.33 | 10.4 | 7.58 | 4.57 |
| 1979, Imperial Valley | 6.5 | 2 | 0.49 | 19 | 0.12 | 3.3 | 6.15 | 1.51 |
| 1964, Niigata | 7.5 | 21 | 0.32 | 11 | 0.28 | 12.2 | 2.9 | 1.65 |
| 1964, Niigata | 7.5 | 21 | 0.32 | 8 | 0.34 | 11.4 | 3.1 | 2.09 |
| 1964, Niigata | 7.5 | 21 | 0.32 | 5 | 0.45 | 10 | 2.86 | 1.82 |
| 1964, Niigata | 7.5 | 21 | 0.32 | 12 | 0.24 | 11.9 | 4.45 | 3.38 |
| 1995, Hyogo-Ken Nanbu | 6.8 | 6.5 | 0.37 | 10 | 1.88 | 12.5 | 14.63 | 1.47 |
| 1964, Niigata | 7.5 | 21 | 0.32 | 5 | 0.35 | 12.7 | 2.79 | 1.01 |
| 1964, Niigata | 7.5 | 21 | 0.32 | 13 | 0.29 | 12.9 | 3.04 | 0.42 |
| 1964, Niigata | 7.5 | 21 | 0.32 | 5 | 0.31 | 14.1 | 17.75 | 8.39 |
| 1964, Niigata | 7.5 | 21 | 0.32 | 11 | 0.24 | 12.1 | 2.11 | 1.27 |
| 1964, Niigata | 7.5 | 21 | 0.32 | 7 | 0.28 | 8.1 | 17.05 | 6.18 |
| 1979, Imperial Valley | 6.5 | 2 | 0.49 | 21 | 0.11 | 1.4 | 4.69 | 0.87 |
| 1979, Imperial Valley | 6.5 | 2 | 0.49 | 25 | 0.11 | 2.2 | 3.52 | 0.47 |
| 1971, San Fernando | 6.4 | 0.5 | 0.68 | 47 | 0.08 | 5.2 | 19.96 | 2.63 |
| 1964, Niigata | 7.5 | 21 | 0.32 | 6 | 0.35 | 0.5 | 5.96 | 0.77 |
| 1995, Hyogo-Ken Nanbu | 6.8 | 6 | 0.38 | 13.4 | 0.94 | 12.5 | 15 | 1.48 |
| 1995, Hyogo-Ken Nanbu | 6.8 | 5.5 | 0.39 | 10 | 1.36 | 15 | 9.79 | 1.47 |
| 1989, Loma Prieta | 7 | 27.2 | 0.2 | 2 | 0.8 | 2.7 | 33.54 | 0.29 |
| 1987, Superstition Hills | 6.6 | 23 | 0.15 | 25 | 0.09 | 3.4 | 41.38 | 0.24 |
| 1999, Chi-Chi | 7.6 | 5 | 0.67 | 13 | 0.18 | 0.5 | 5.7 | 0 |
| 1999, Chi-Chi | 7.6 | 5 | 0.67 | 13 | 0.18 | 0.75 | 6.6 | 0.1 |
| 1999, Chi-Chi | 7.6 | 5 | 0.67 | 13 | 0.18 | 0.75 | 7.9 | 0.17 |
| 1999, Chi-Chi | 7.6 | 5 | 0.67 | 13 | 0.18 | 0.75 | 9 | 0.23 |
| 1999, Chi-Chi | 7.6 | 5 | 0.67 | 13 | 0.18 | 0.75 | 15 | 0.29 |
| 1964, Niigata | 7.5 | 21 | 0.32 | 6 | 0.35 | 0.5 | 2.29 | 1.38 |
| 1964, Niigata | 7.5 | 21 | 0.32 | 5 | 0.31 | 14.1 | 4.55 | 6.67 |
| 1964, Niigata | 7.5 | 21 | 0.32 | 11 | 0.27 | 12 | 3.98 | 1.83 |
| 1995, Hyogo-Ken Nanbu | 6.8 | 8 | 0.34 | 14.6 | 1.98 | 16 | 8.45 | 0.41 |
| 1964, Niigata | 7.5 | 21 | 0.32 | 6 | 0.29 | 7.9 | 17.05 | 5.39 |
| 1964, Niigata | 7.5 | 21 | 0.32 | 11 | 0.27 | 12.1 | 2.97 | 1.49 |
